# Comprehensive exercise program based on optimal physiotherapy for asthma-related quality of life: a systematic review and network meta-analysis

**DOI:** 10.3389/fspor.2025.1738390

**Published:** 2026-01-20

**Authors:** Danli Song, Junchao Zhang, Zeyang Zhao, Xinmiao Feng, Linlin Zhao, Jinzhao Yang, Xie Jing

**Affiliations:** 1 Sports Coaching College, Beijing Sport University, Beijing, China; 2 Hebei Institute of Mechanical and Electrical Technology, Xingtai, Hebei, China; 3 China Wushu School, Beijing Sport University, Beijing, China; 4 Department of Police Tactics and Techniques, Hebei Public Security Police Vocational College, Shijiazhuang, China

**Keywords:** asthma, chronic disease, evidence-based medicine, exercise, quality of life (QoL)

## Abstract

**Background:**

Exercise is an effective treatment for asthma, but there is still ongoing debate regarding the most beneficial form of exercise. This study used data from randomized controlled trials to compare and rank the types of exercise that improve asthma-related quality of life (QOL) in patients [total score and four subdomain scores (symptoms, activity limitations, emotional functions, and environmental stimulants)].

**Method:**

This study was meticulously conducted using a rigorous methodology. We included randomized controlled trials of 10 types (two major categories: single-mode exercise and comprehensive-mode exercise) of exercise for the interventional treatment of asthmatics (≥16 years old). The effect size measures were standardized mean differences (SMDs) with 95% credible intervals (CrIs). The confidence of evidence was examined using Confidence in network meta-analysis. The study protocol has been registered with PROSPERO under registration number CRD420251110553.

**Result:**

We identified 1,237 citations based on 35 studies involving 3,946 participants. Compared with the control group, all comprehensive-mode exercises (yoga, aerobic combined with breathing or resistance exercise) significantly improved the total score of asthma-related QOL (and four subdomain scores) among participants, and the SMDs [95% Credible Interval (CrI)] ranged between 2.26 (1.63 to 2.90) for Yoga to 1.06 (0.01 to 2.11) for high-intensity interval training (HIIT). Notably, HIIT demonstrated promising trends in improving asthma-related QOL compared to moderate-intensity aerobic exercises (*P*-score = 0.58). While Buteyko (*P*-score = 0.48) ranked first among various breathing exercises, the differences in efficacy between these methods were mostly small or uncertain. exercise prescription were significant factors affecting the network meta-analysis results.

**Conclusion:**

Various comprehensive exercise modes are the best way to improve asthma-related QOL in patients. The exercise period will affect the effectiveness of the rehabilitation program.

**Systematic Review Registration:**

https://www.crd.york.ac.uk/PROSPERO/view/CRD420251110553, PROSPERO CRD420251110553.

## Introduction

Asthma, a chronic inflammatory airway disease, exhibits a globally increasing trend in both incidence and mortality rates (1). Projections suggest that the number of asthma patients worldwide could escalate to 400 million by 2025 (2). The symptoms associated with asthma encompass a spectrum of manifestations, including breathlessness, chest tightness, coughing, and airflow obstruction (3), thereby significantly impeding the daily life and routine activities of affected individuals. Despite the proven efficacy of pharmaceutical interventions in asthma management (4), numerous patients fail to achieve complete disease control due to treatment inadequacy and non-compliance (5). There are also concerns about potential adverse effects associated with long-term medication use, specifically corticosteroids (6). Consequently, there is growing support for non-pharmacological respiratory rehabilitation strategies (7).

As a critical part of non-pharmacological pulmonary rehabilitation, regular exercises have demonstrated efficacy in ameliorating pulmonary function (7), physical fitness (8), cardiovascular wellbeing, airway hyperreactivity (9) and symptoms (10) in asthmatic patients. Moreover, these adaptive changes positively correlate with asthma control and quality of life (QOL) (11). Though most patients may have experienced exercise-induced bronchoconstriction (EIB), current guidelines recommend mitigating the risk of EIB by using medications and pre-exercise warm-ups based on distinct asthma phenotypes (2).

Numerous meta-analyses exist to date regarding exercise treatment for asthmatic adults. Wu et al. demonstrate that regular and continuous aerobic exercise benefits asthma patients regarding QOL (12). However, they failed to consider the potential benefits of additional components included in some programs, such as breathing or strength training. Similarly, several studies have examined yogic ventilation techniques constituting a comprehensive yoga session (13, 14). Typically, evidence-based guidelines do not recommend high-intensity exercise (2), but a review reported the feasibility of High-Intensity Interval Training (HIIT) for asthma (15). Moreover, some empirical studies with limited sample sizes suggest that HIIT may provide additional benefits for asthma, which also needs further exploration (16, 17). Breathing exercises have gained recognition in pulmonary rehabilitation guidelines due to their potential to enhance asthma-related QOL (2). However, most studies have not investigated potential differences in therapeutic effects for asthma offered by various ventilatory techniques (18, 19), each requiring distinct breathing patterns. Analyzing the benefits of each ventilation technique can guide the development of the new intervention. An inherent limitation of traditional meta-analyses is their inability to discern a hierarchy among various interventions. Enhancing the specificity of different exercise regimens allows us to integrate the results of both direct and indirect comparisons using a network meta-analysis (NMA) (20).

There are three reasons for treating asthma patients: to prevent mortality, to reduce the probability of future morbidity, and to improve patient well-being (11, 21). Most conventional clinical measures of asthma control and severity are predominantly based on the patient's airway status, which primarily serves to assess the achievement of the first two objectives. However, correlational studies indicate that assessments of clinical status do not fully capture the evaluation of patient well-being (22, 23). As a distinct component of the overall asthma health status, Quality of Life (QOL) is a subjective criterion influenced not only by the severity and control of the disease itself but also significantly by non-physiological factors (24–26), such as environmental challenges and emotional distress (25, 27). Therefore, an independent evaluation of QOL is necessary, underscoring the importance of a patient-centered, holistic management strategy. Consequently, this research uses NMA to assess and rank the impact of various exercise regimes on asthma-related QOL in adult patients.

## Method

This study was conducted strictly by the Preferred Reporting Items for Systematic Reviews and Meta-analyses (PRISMA) statement (Supplementary Information S1-S2) (28). The study protocol has also been registered with PROSPERO under registration number CRD420251110553.

### Data source and search

The English electronic databases were searched systematically for this study as follows: PubMed, Web of Science, Embase, EBSCO, and Cochrane Library from their inception date to March 30, 2025. The following search syntax was utilized: (“asthma”) AND (“pulmonary rehabilitation” OR “physical exercise” OR “aerobic exercise” OR “breathing exercise” OR “pranayama” OR “Buteyko” OR “diaphragm” OR “Papworth” OR “ventilatory muscle training” OR “interval training” OR “yoga”) NOT (“children”) (Supplementary Information S3). Besides, we manually searched all review articles related to the physical rehabilitation of asthma and traced additional possible studies by reviewing their reference lists.

### Study selection

**Figure 1:**
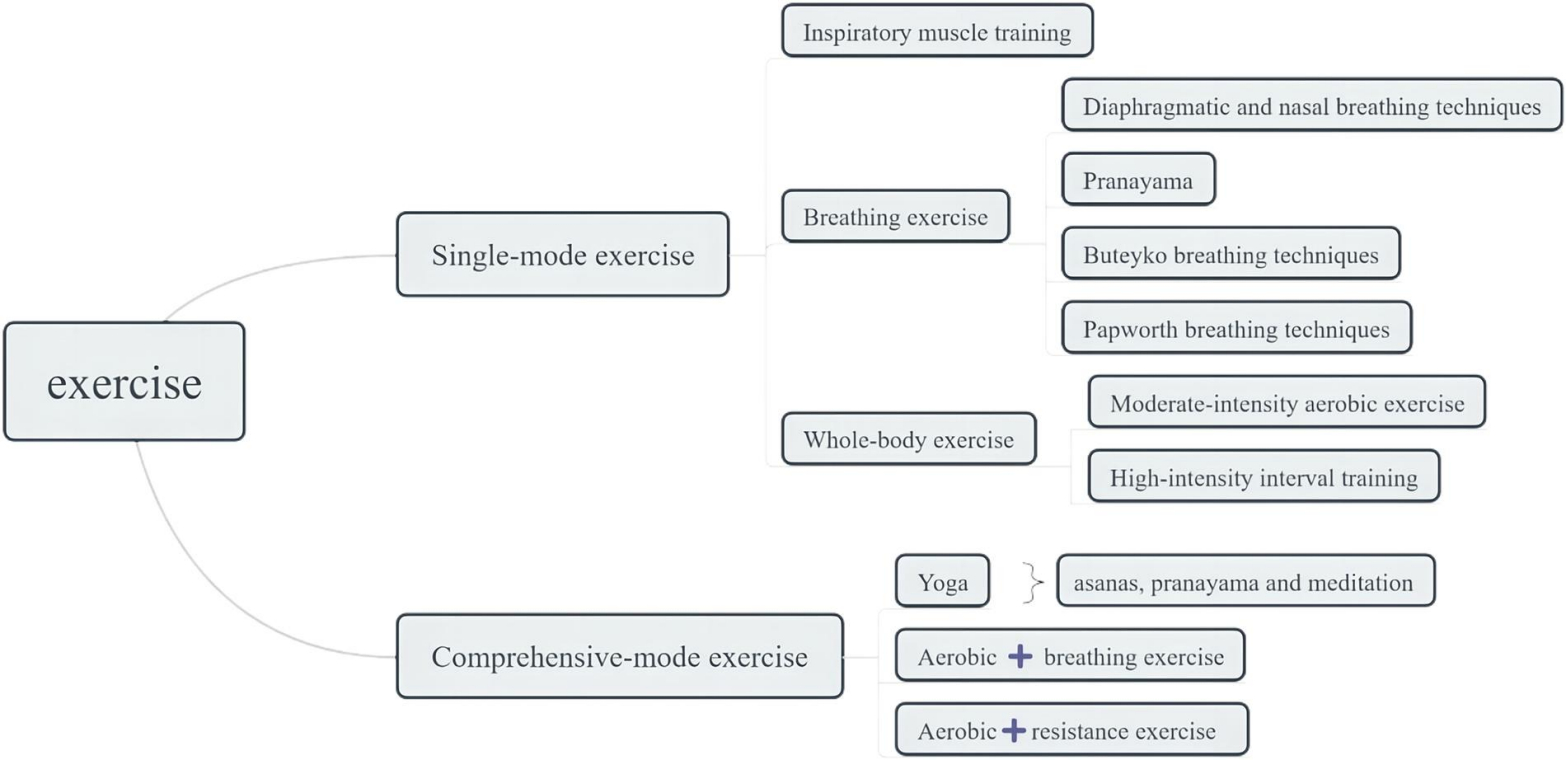
Classification of exercise types.

The selection standards were based on the PICOS approach (participants, interventions, comparators, outcomes, and study design). The included participants were clinically diagnosed with asthma; their mean age was 16 years and above, and there was no restriction on obesity or overweight. According to the training content, the rehabilitation exercises were divided into ten types, as depicted in Figure 1 and Table 1 (Supplementary Information S4). The control group included health education or usual care. Besides, the comparator chosen for head-to-head studies can be any of the ten exercise types. The study included the outcomes measured by QOL, encompassing total score or subdimension scores across four domains: symptoms, activity limitation, emotional function, and environmental stimuli (29). This study used a randomized controlled trial (RCT) as a study design. Furthermore, we excluded the following types of studies: asthmatic participants with additional comorbidities, acute intervention, meeting abstracts, book chapters, commentaries to articles, and study protocols.

**Table 1:** Definition of exercise types and non-exercise group.

Abbreviation	Full name	Definitions
aerobic	Moderate-intensity aerobic exercise	Aerobic exercise is performed by activities for extended periods of time. e.g., walking, bicycle, treadmill training etc.
HIIT	High-intensity interval training	High-Intensity Interval Training (HIIT) is a exercise training method characterized by alternating short periods of high-intensity exercise with rest or low-intensity exercise. HIIT training includes brief high-intensity exercises (such as sprinting or fast cycling) followed by relatively short periods of rest or low-intensity exercise (such as walking or slow cycling). This cycle of alternating intensity levels continues for a set period of time, with training sessions typically being short but highly effective.
CON	Control group	Non-exercise intervention, usual care, or asthma education
breathing	Diaphragmatic and nasal breathing exercise	Diaphragmatic breathing (DB) is slow and deep breathing that affects the brain and the cardiovascular, respiratory, and gastrointestinal systems through the modulation of autonomic nervous functions.
pranayama	Pranayama breathing exercise	Pranayama is a breathing control technique in yoga. The main function of pranayama is to promote smooth breathing and unity of body and mind for practitioners.
Buteyko	Buteyko breathing exercise	The late Professor Konstantin Buteyko was a Russian physiologist who gave his name to a novel treatment approach that is currently being applied to patients with asthma in a number of countries. The approach varies in some details in different countries and with different practitioners, but essentially consists of a package of breathing therapy, relaxation techniques and exercises combined with advice and education about medication use, nutrition and general health.
papworth	Papworth breathing exercise	An integrated breathing and relaxation technique known as the Papworth method has been implemented by physiotherapists since the 1960s for patients with asthma and dysfunctional breathing.
aerobic_ breathing	Aerobic exercise combined with Breathing training	Aerobic exercise combined with Breathing training
aerobic_ resistance	Aerobic exercise combined with resistance training	Aerobic exercise combined with resistance training. resistance training designed to improve the strength, power, endurance and size of skeletal muscles.
IMT	Inspiratory muscle training	Inspiratory Muscle Training (IMT) is a training method that can improve inspiratory muscle strength in ICU patients. IMT is conducted using a handheld device that adjusts inspiratory resistance during the inspiratory phase to train the inspiratory muscles of patients.
Yoga	-	Mainly a series of methods for self-cultivation, including body-adjusting asanas (refer to yoga asana collection), breathing-adjusting breathing methods, and mind-adjusting meditation, etc., to achieve the unity of body and mind.

### Data abstraction and quality assessment

The relevant articles obtained from the aforementioned electronic databases were stored in the EndNote X9 reference manager, and two reviewers (JC and DL) reviewed and selected the retrieved articles based on the reference criteria mentioned above. Subsequently, relevant data was extracted from the qualified articles. Information extracted included publication information (author and year), participants (sample size, gender, BMI, age, asthma status, and measurement), experimental design, interventions (exercise program, period, duration, frequency and intervention conditions), comparator, and outcomes. The outcome measure was QOL (total score and subdimension scores), which was assessed using the asthma QOL questionnaire (AQLQ) (30), Mini AQLQ (29), or St George's Respiratory Questionnaire (SGRQ) (31). When the study data was not enough for meta-analysis, we tried to contact the corresponding author through email to request supplementary data. The methodological quality of the included articles was evaluated by two reviewers (JC and DL) using the Physiotherapy Evidence Database (PEDro) scale (32). A total of 11 items are incorporated in the PEDro scale, comprising 3 items derived from the Jadad scale and nine items from the Delphi list. The PEDro scale score is utilized to assess the quality of RCTs, with scores ranging from 0 (low quality) to 10 (high quality). A score of 6 or greater represents high-quality research. The first item on the PEDro scale (eligibility criteria specified) is used to establish external validity; thus, the score is not included in the total score. Any disagreement during the above process was resolved by a review group within the team through the process of reaching a consensus and engaging in arbitration.

### Statistical analysis

The research employed network meta-analytic techniques via R statistical software (v3.6.3), specifically using the netmeta package to merge direct and indirect evidence in a frequency model (33). Effect sizes were reported as standardized mean differences (SMD) with 95% credible intervals (CrI). A random-effects network meta-analysis (NMA) model was applied to synthesize the effect estimates. In the network plot, edge width denotes direct comparisons, and node size reflects study sample size. The edge width corresponds to the number of studies providing direct comparison between interventions. When a direct connection was absent between physical activity types, indirect comparisons were conducted via network meta-analysis. The standardized mean differences (SMDs) with 95% CrIs for all pairwise comparisons are summarized in a league table, with effects vs. a control detailed in an accompanying forest plot. Physical activity modalities were ranked by *P*-scores according to their efficacy in improving asthma-related QOL. The *P*-score is bounded between 0 and 1, with a higher value denoting a superior enhancement in aerobic capacity (34). Heterogeneity across studies was assessed using the tau-squared (*τ*^2^) statistic and its associated *p*-value (35, 36). Larger *τ*^2^ values and smaller *p*-values indicate greater heterogeneity, whereas smaller *τ*^2^ values and larger *p*-values suggest lower heterogeneity. Furthermore, the I^2^ statistic, ranging from 0% to 100%, was employed to quantify the heterogeneity among study outcomes. Heterogeneity was classified as low (I^2^ < 25%), moderate (25%–50%), or high (I^2^ > 75%). An I^2^ value exceeding 50% was considered indicative of substantial heterogeneity. Global and local methods were employed to evaluate network inconsistency, with the design-by-treatment interaction model applied to assess global heterogeneity (37). Local inconsistency was evaluated by applying the node-splitting method within the R netmeta package (38). Network meta-regression (R gemtc package) was employed to investigate sources of heterogeneity, including publication year, sample size, mean age, sex proportion, and exercise parameters (duration, frequency, and time per session). Adjusted funnel plots, analyzed in conjunction with Egger's test (*p* < 0.05 indicating significance), were used to evaluate potential publication bias. To assess robustness, the network meta-analysis was repeated after excluding high-risk-of-bias studies (see Supplementary Information S6 for detailed methods).

## Result

### Study characteristics and quality assessment

**Figure 2:**
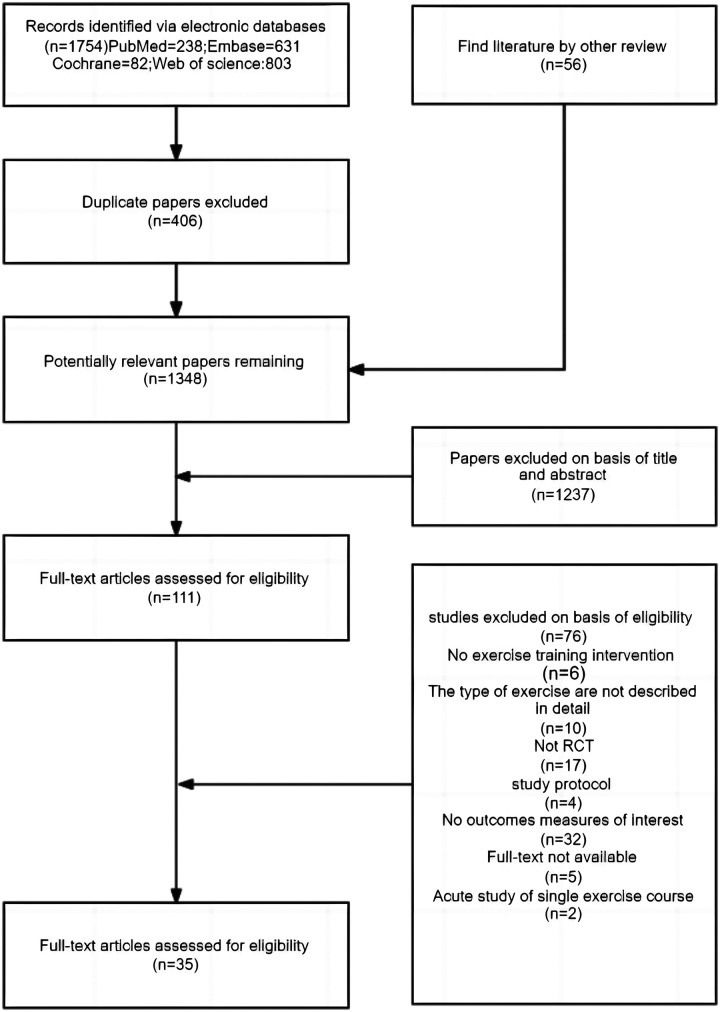
Search terms and outcomes

Figure 2. depicts the search process for systematic reviews. After excluding 1,237 reports based on the title and abstract, 111 full-text articles were retrieved. While examining the full texts, we selected and included 35 studies with 3,946 participants, 1,953 (49.5%) of whom were male, and 1,993 (50.5%) were female. The sample size ranged from 20 to 255, with a mean year of 43.42 (SD 8.89). The exercise period ranged from 4 to 52 weeks (mean period 15.132 weeks, SD 10.962), the frequency of exercise training per week ranged from 2 to 7 (mean frequency 3.943, SD 1.982), and the total time of the single session ranged from 15 to 90 min (mean time 40.286 min, SD 18.745) (Table 2 and Supplementary Information S7). The intervention primarily comprised two conditions. The first was a group-based program, consisting of face-to-face exercise sessions conducted under the guidance of a trained instructor. The second was a home-based program, where participants self-directed their exercises following a plan provided by a professional. Prior to the home-based program, all studies provided a one- to two-week training protocol to ensure correct technique and program consistency. Some studies incorporated compliance-ensuring strategies for the home-based condition, such as regular reminders via phone or internet message, exercise logs for self-monitoring, or periodic follow-ups by researchers. In contrast, others did not describe any supervision methods to ensure participant compliance (Table 2). The PEDro scale was used to determine the quality of the included study, with results showing an average score of 8.2 ± 1.41 and indicating a generally high methodological quality. Only one study had scores below the predetermined threshold of 5 points (Supplementary Information S8).

**Table 2:** Characteristics of included studies.

Study	Sample size		Asthma status and measurement	Exercise program		Intervention conditions
	Exercise group	Control group				
Turan et al. 2019	n 56 M/F 7/49	n 56 M/F 11/45	mild to moderate asthma ≥ 6 months (FEV_1_ >80%)	yoga	twice a week for 6 weeks one session for 70 min [pranayama, asanas (poses) and relaxing]	Group-based
Yüce et al. 2020	n 25 M/F 3/22	n 25 M/F 2/23	severe asthma ≥ 6 months (FEV_1_ ≤ 60%)	pranayama	20 min once daily for 1 month	home-based under supervision
Thomas et al. 2009	n 94 M/F 42/52	n 89 M/F 29/60	moderate asthma ≥ 6 months (FEV_1_ >80%)	Papworth	·Study attendances for both groups consisted of 3 sessions, an initial 60 min small group session (2–4subjects) followed by 2 individual sessions of 30–45 min with 2–4 weeks between attendances. ·10 min once daily for 1 month at home. ·The entire study lasted for 1 year, and 2 tests were administered at the end of the first month and the final month, respectively (diaphragmatic and nasal breathing techniques, similar to the Papworth)	home-based unspecified supervision
França-Pinto et al. 2014	group 1: n 22 M/F 5/17 group 2: n 21 M/F 4/17		moderate or severe persistent asthma	group 1: aerobic_breathing group 2: pranayama	*aerobic exercise* twice a week for 12 weeks one session lasted 35 min (5 min of warm-up,25 min of aerobic training and 5 min of cool-down) Aerobic exercise (indoor treadmill) was initiated at 60% of VO2max in the first 2 weeks and then increased to 70% VO2 max *pranayama breathing exercise* twice a week for 12 weeks one session lasted 30 min	Group-based
Vempati et al. 2009	n 29 M/F 13/16	n 29 M/F 13/16	mild to moderate asthma ≥ 6 months (FEV_1_ > 60%)	yoga	5 times a week for 12 weeks one session lasted 90 min The program consisted of lectures and practical sessions on asanas (postures), pranayamas (breathing techniques), kriyas (cleansing techniques), meditation and shavasana (a relaxation technique).	home-based under supervision
Türk et al. 2002	group 1: n 14 M/F 4/10 group 2: n 7 M/F 3/4	n 10 M/F 1/9	obesity with suboptimal controlled asthma (FEV_1_ > 80%)	group 1: HIIT group 2: HIIT with Internet based self-management	three times a week for 12 weeks (warming up lasted for 10.5 min, reach 7 at the 10-grade Borg scale; stretching exercises lasted for 10 min; interval high-intensity training, [the first seven weeks: 3 sets of 4 × 45s session of 90% VO2max], [the last four weeks: 6 sets of 4 × 45s session of 90% VO2max];cooling down for 5 min)	Group-based
Ma et al. 2014	n 165 M/F 49/116	n 165 M/F 165/0	obesity with uncontrolled persistent asthma	aerobic	at least 150 min/wk of moderate-intensity a 12-month lifestyle intervention (brisk walking)	Transition from Group-based (4-months) to Tele-supervised (8-months)
Malarvizhi et al. 2018	n 125 M/F 70/55	n 125 M/F 69/56	mild to moderate asthma (FEV_1_ > 60%)	yoga	once a day for 6 months one session lasted for 30 min [basic asanas (postures) and pranayama]	home-based under supervision
Bidwell et al. 2012	n 12 M/F 0/12	n 8 M/F 0/8	clinical mild-to-moderate asthma	yoga	·a supervised yoga programme: twice a week for 10 weeks one session for 60 min ·a home yoga programme: once a week for 10 weeks one session for 60 min [relaxation/deep breathing, asanas (postures), meditation and relaxation]	Group-based with home-based
Bruton et al. 2017	group 1: n 261 M/F 164/97 group 2: n 132 M/F 41/91	n 262 M/F 98/164	clinical asthma ≥ 12 months (FEV_1_ > 60%)	group 1: DVD and booklet breathing training group 2: Face-to-face breathing training	3 times a week for 12 months one session for 40 min	group 1: home-based under supervision group 2: Group-based
Silva et al. 2022	group 1: n 27 M/F 4/23 group 2: n 28 M/F 5/23		moderate to severe persistent asthma	group 1: HIIT group 2: aerobic	*high-intensity interval training* twice a week for 12 weeks, one session lasted 40 min [5 min of warm-up, 30 min of exercise (15 work interval of 30s interspersed with 30s of recovery,intensity from 80 to 140 Wmax), and 5 min of cool down] *aerobic exercise* twice a week for 12 weeks, CLE sessions lasted 40 min (5 min of warm-up, 30 min of Constant-Load cycle ergometer exercise at intensity from 70 to 90 Wmax, and 5 min of cool down)	Group-based
Cooper et al. 2003	group 1: n 30 M/F 15/15 group 2: n 30 M/F 16/14	n 29 M/F 18/11	stable asthma (FEV_1_ > 50%)	Buteyko	one session for at least 15 min twice a day for 6 months	home-based under supervision
Duruturk et al. 2018	n 20 M/F 6/14	n 18 M/F 1/17	mild to moderate persistent asthma (FEV_1_ >80%)	IMT	30 breaths using a patient-specific threshold pressure device, twice daily for 6 wk at 50% maximal inspiratory pressure	Group-based
Evaristo et al. 2020	group 1: n 25 M/F 8/17 group 2: n 29 M/F 7/22		moderate-to-severe persistent asthma (FEV_1_ >60%)	group 1: pranayama group 2: aerobic	*pranayama breathing exercise* twice a week for 12 weeks one session lasted for 40mins *aerobic training* twice a week for 12 weeks one session lasted for 40mins (5 min of warm-up, 30 min of indoor treadmill at 60% HRmax, and 5 min of cool-down)	home-based unspecified supervision
Sarah A. Hiles et al. 2021	n 15 M/F 6/9	n 9 M/F 4/5	severe asthma (FEV_1_ >60%)	yoga	twice times a week for 16 weeks one session lasted for 75 min (Asana, Pranayama and Meditation)	Group-based
Lage et al. 2021	n 20 M/F 6/14	n 19 M/F 4/15	asthma ACT > 18 points	IMT	5 days a week for 8 weeks, consisting of six sets of 30 breaths per day with a training load ⩾50% of maximal inspiratory pressure	home-based unspecified supervision
Mendes et al. 2010	group 1: n 34 M/F 5/29 group 2: n 45 M/F 10/35		mild to severe persistent asthma ≥ 6 months (FEV_1_ > 80%)	group 1: aerobic_breathing group 2: pranayama	*pranayama breathing exercise* yoga breathing exercises (Kapalabhati, Uddhiyana, Agnisara) a 30-min session was performed twice a week for 3 months *aerobic exercise* 30 min per session twice a week for 3 months at intensity from 60 to 70% VO2max	home-based unspecified supervision
Prem et al. 2012	group 1: n 29 M/F 16/23 group 2: n 36 M/F 17/19	n 40 M/F 14/26	mild to moderate asthma > 6 months	group 1: buteykogroup 2: pranayama	*buteyko breathing exercise* one 15-min session twice daily for 3 months *pranayama breathing exercise* one 15-min session twice daily for 3 months (diaphragmatic breathing, thoracic breathing, upper lobe breathing and full yogic breathing progressing to right nostril breathing, left nostril breathing and alternate nostril breathing)	home-based under supervision
Sabina et al. 2005	n 29 M/F 5/24	n 33 M/F 11/22	mild to moderate asthma > 6 months	yoga	twice-weekly 90-minute yoga sessions for 4 weeks including 15 asanas (postures), pranayama (breathing), and dhyana (meditation)	Group-based
Thomas et al. 2012	n 17 M/F 4/13	n 16 M/F 3/13	diagnosed and currently treated asthma	breathing	one session for at least 15 min twice a day for 6 months (diaphragmatic breathing)	home-based unspecified supervision
Toennesen et al. 2017	group 1: n 71 M/F 16/55 group 2: n 31 M/F 7/24	control 1: n 32 M/F 8/24 control 2: n 32 M/F 8/24	ACQ score 12 of 1.0 or more	group 1: HIIT group 2: HIIT with diet control	3 times a week for 8 weeks Each session included 10-minute warm-up at a low intensity followed by either two, three, or four 5-minute intervals (during weeks 1-2, 3-5, and 6-8, respectively). Each 5-minute interval consisted of 5 consecutive 1-minute intervals divided into 30, 20, and 10 s at an intensity corresponding to less than 30%, less than 60%, and more than 90% of maximal intensity.	Group-based
Manocha et al. 2002	n 21 M/F 10/11	n 26 M/F 11/15	asthma remained poorly control (FEV_1_ > 70%)	yoga	a 2 h session once a week for 4 months	Group-based with home-based
Andreasson et al. 2022	n 84 M/F 36/58	n 99 M/F 35/64	Moderate to severe asthma remained poorly control (FEV_1_ > 65%)	breathing	one session for at least 15 min twice a day for 6 months (in brief, nasal inhalation; breathing from diaphragm and lower chest; normalization of the tidal volume; shoulder, neck, tongue, and jaw relaxation; exhalation prolongation and/or breath-hold technique (if elevated respiration frequency) and starting at relaxed body position progressing to use during physical activity)	home-based unspecified supervision
Zaryyab et al. 2021	group 1: n 10 M/F 5/5 group 2: n 10 M/F 6/4		asthma remained poorly control > 12 months	group 1: papworth group 2: buteyko	*papworth breathing exercise* independent sessions five days per week for a period of 3 months *buteyko breathing exercise* independent sessions five days per week for a period of 3 months	home-based under supervision
Gonçalves RC et al. 2008	group 1: n 10 M/F 3/7 group 2: n 10 M/F 4/6		asthma remained control (FEV_1_ > 65%)	group 1: aerobic_breathing group 2: pranayama	*pranayama breathing exercise* twice a week for three months one session lasted 30 min *aerobic exercise* twice a week for three months one session lasted 30 min (treadmill running) with the intensity of 70% HRmax	Group-based
Refaat et al. 2015	n 38 M/F 17/21	n 30 M/F 14/16	moderate to severe asthma (FEV_1_ > 60%)	aerobic_resistance	3 exercise sessions every week for 6 weeks one session lasted 30 min 10 min warm-up included slow walking and stretching Each aerobic circuit was comprised of cycle ergometry training, step ups, wall squats and upper limb endurance training (60%–80% HRmax), followed by a 5 min cooling down period that comprised of a 150 m slow walk on a treadmill at 40% of HRmax	Group-based
Scott et al. 2013	n 13 M/F 6/7	n 15 M/F 7/8	obesity with uncontrolled persistent asthma	aerobic_resistance	3 times a week for 12 weeks one session for 60 min	Group-based
Holloway et al. 2023	n 46 M/F 18/28	n 39 M/F 18/21	patients are registered on the practice asthma database	papworth	5 sessions of treatment by papworth one session lasted for 60 min	
Coulson et al. 2021	n 45 M/F 12/33	n 45 M/F 35/10	olders with persistent asthma	pranayama	twice per day (10 min per session) for one month (pranayama, diaphragmatic breathing and pursed lip breathing)	home-based unspecified supervision
SODHI et al. 2009	n 60 M/F 34/26	n 60 M/F 37/23	mild to moderate asthma (FEV_1_ > 70%)	yoga	twice a day for 8 weeks one session lasted 45 min pranayamas (deep breathingexercises), kapalabhati (cleaning breath), bhastrika (rapid and deep respiratory movements like that of the bellows), ujjayi (loud sound producing pranayama) and sukha purvaka pranayama (easy comfortable breathing)	Group-based with home-based
SODHI et al. 2014	n 60 M/F 34/26	n 60 M/F 37/23	asthma remained well controlled	yoga	twice a day for 8 weeks one session lasted 45 min	Group-based with home-based
Cowie et al. 2008	n 60 M/F 34/26	n 64 M/F 14/50	moderate to severe persistent asthma (FEV_1_ > 60%)	buteyko	5 sessions each week for 6 weeks	home-based unspecified supervision
Agnihotri et al. 2018	n 125 M/F 125/0	n 130 M/F 130/0	mild-to-moderate persistent asthma	yoga	30 min per day, 5 days in a week for 6 months (Asanas, Pranayama and Meditation)	Group-based
Meyer et al. 2015	n 13 M/F 5/8	n 8 M/F 5/3	mild-to-moderate persistent asthma (FEV_1_ > 60%)	aerobic_breathing	A 15-min warm-up period of walking at different speeds accompanied by light exercises of different muscle groups was followed by endurance and circuit training including upper and lower extremities for 30 min (> 60% of the maximum heart rate). using diaphragmatic breathing and pursed lip breathing to improve ventilation.	Group-based
Freitas et al. 2017	n 125 M/F 125/0	n 130 M/F 130/0	obesity with well controlled asthma (FEV_1_ > 60%)	aerobic_resistance	incorporated aerobic and resistance exercises (two sessions per week, 3 months) into the weight-loss program. Aerobic training intensities were based on 50%–75% of peak VO2. Patients performed resistance training for major muscle groups, used an accelerometer, and completed a physical activity diary	Group-based
Holloway et al. 2023	n 46 M/F 18/28	n 39 M/F 18/21	Asthma (FEV_1_ < 80%)	papworth	at least once a day, 12 months one session lasted for 60 min Assessments took place at baseline, post-treatment (approximately 6 months after baseline) and at 12 months.	home-based under supervision

F “female”; M “male”; ACQ “asthma control Questionnaire”; FEV_1_ “ forced expiratory volume in one second in litre/% of predicted”; breathing “diaphragm breathing exercsie”; papworth “Papworth breathing exercise”; IMT “inspiration muscle training”; buteyko “Buteyko breathing exercise”; aerobic “moderate-intensity aerobic exercise”; HIIT “High-intensity interval training”; pranayama “Pranayama exercise”; aerobic_breathing “aerobic combined with breathing exercise”; aerobic_resistance “aerobic combined with resistance exercise”; Group-based “a structured, group exercise program conducted under the guidance of professional personnel”; home-based “Self-directed exercise programs in locations of the patient's choice”; under supervision “Home-based programs with adherence strategies, such as remote supervision (regular phone calls, internet-based reminders), self-reporting (daily exercise logs), or in-person follow-ups”; unspecified supervision“Home-based programs without described adherence strategies”.

### Network meta-analysis

**Figure 3:**
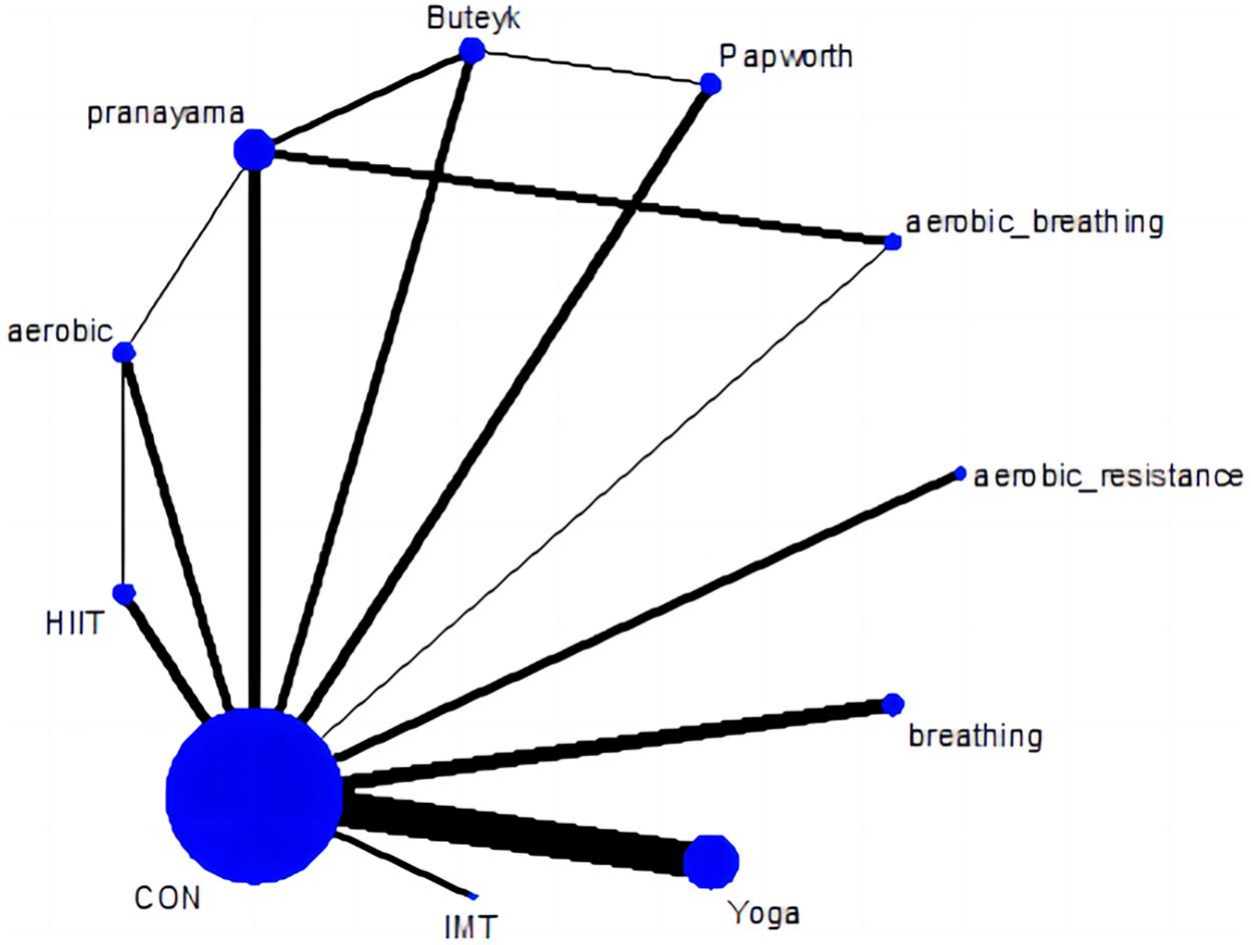
Network plot of asthma QOL total score.

**Figure 4:**
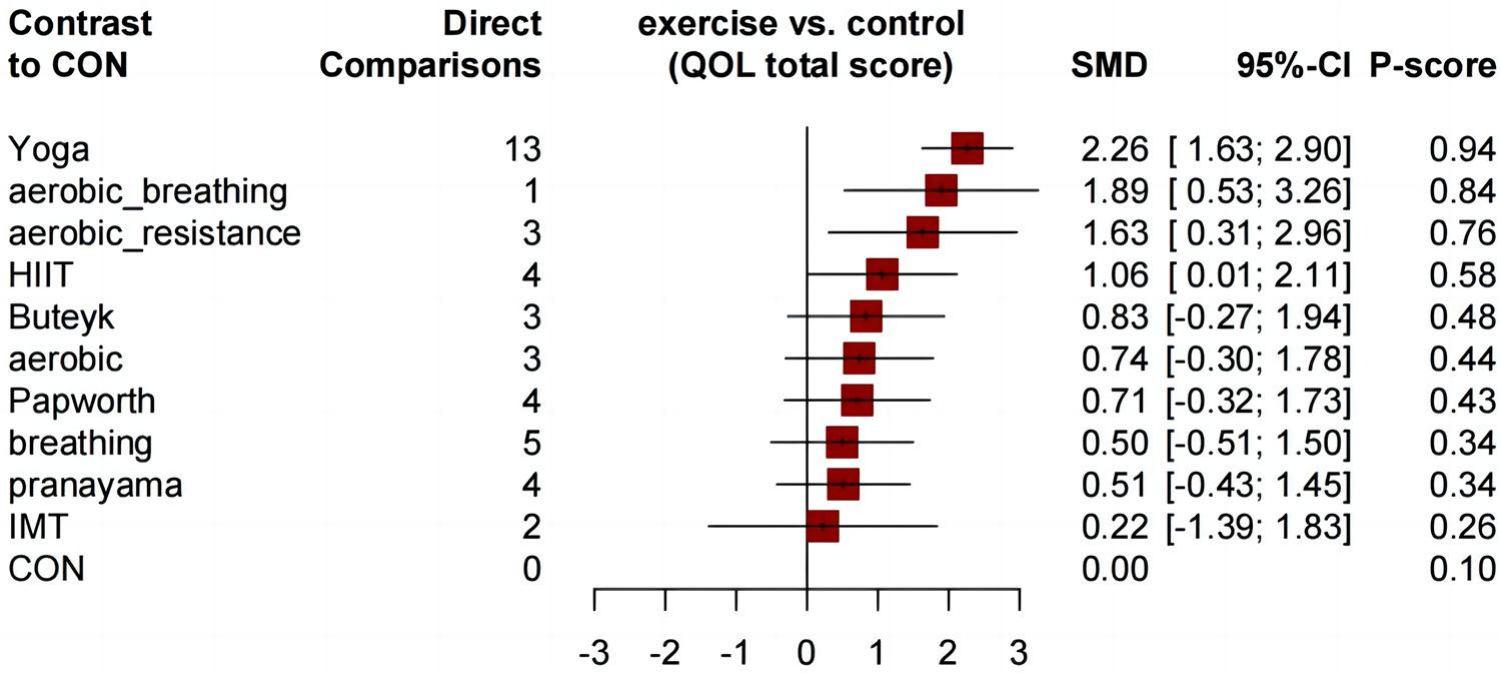
Forest plot change in effect of asthma QOL total score.

Figure 3. presents a network diagram showing the comparisons of qualified asthma-related QOL total score (each QOL subdimension score network diagrams are shown in Supplementary Information S5); all exercise methods were compared with the control group at least once. Compared with the control group, all comprehensive-mode exercises significantly improved the asthma-related QOL total score of participants, and the SMDs [95% Credible Interval (CrI)] ranged between 2.26 (1.63 to 2.90) for Yoga to 1.06 (0.01 to 2.11) for HIIT (Figure 4 and Table 3), and Yoga ranks first (*P*-score = 0.94). Compared to moderate-intensity aerobic exercises (*P*-score = 0.58), HIIT has shown better trends in improving asthma QOL. Although Buteyko (*P*-score = 0.48) is ranked first among various breathing exercises, the differences between the efficacy of these methods are mostly small or uncertain.

**Table 3:** League table for changes in asthma-related QOL total score with various exercises.

Yoga	.	.	.	.	.	.	.	.	.	**2.26** **(1.63; 2.90)**
0.37 (−1.14; 1.87)	aerobic_breathing	.	.	.	.	.	.	**1.74 (0.40; 3.08)**	.	0.78 (−1.59; 3.15)
0.63 (−0.84; 2.10)	0.26 (−1.64; 2.17)	aerobic_resistance	.	.	.	.	.	.	.	**1.63** (**0.31; 2.96)**
1.20 (−0.03; 2.43)	0.83 (−0.87; 2.54)	0.57 (−1.12; 2.26)	HIIT	.	0.62 (−1.63; 2.87)	.	.	.	.	0.98 (−0.17; 2.14)
**1.43** (**0.16; 2.70)**	1.06 (−0.57; 2.70)	0.80 (−0.93; 2.52)	0.23 (−1.29; 1.74)	Buteyko	.	−0.06 (−2.41; 2.30)	.	0.47 (−1.12; 2.05)	.	0.96 (−0.33; 2.25)
**1.53** (**0.31; 2.74)**	1.16 (−0.48; 2.79)	0.90 (−0.79; 2.58)	0.33 (−0.99; 1.64)	0.10 (−1.38; 1.58)	aerobic	.	.	0.44 (−1.81; 2.69)	.	0.76 (−0.52; 2.05)
**1.55** (**0.35; 2.76)**	1.19 (−0.50; 2.87)	0.92 (−0.75; 2.60)	0.35 (−1.11; 1.82)	0.13 (−1.22; 1.48)	0.03 (−1.43; 1.48)	Papworth	.	.	.	0.67 (−0.44; 1.78)
**1.77** (**0.58; 2.95)**	1.40 (−0.29; 3.09)	1.14 (−0.52; 2.80)	0.57 (−0.88; 2.02)	0.34 (−1.15; 1.83)	0.24 (−1.20; 1.68)	0.21 (−1.22; 1.65)	breathing	.	.	0.50 (−0.51; 1.50)
**1.75** (**0.62; 2.88)**	**1.38** (**0.20; 2.57)**	1.12 (−0.50; 2.74)	0.55 (−0.83; 1.93)	0.32 (−0.93; 1.57)	0.23 (−1.04; 1.49)	0.20 (−1.16; 1.55)	−0.02 (−1.39; 1.36)	pranayama	.	0.94 (−0.18; 2.07)
**2.04** (**0.31; 3.77)**	1.67 (−0.44; 3.78)	1.41 (−0.68; 3.49)	0.84 (−1.08; 2.76)	0.61 (−1.34; 2.56)	0.51 (−1.40; 2.43)	0.48 (−1.42; 2.39)	0.27 (−1.62; 2.17)	0.29 (−1.58; 2.15)	IMT	0.22 (−1.39; 1.83)
**2.26** (**1.63; 2.90)**	**1.89** (**0.53; 3.26)**	**1.63** (**0.31; 2.96)**	**1.06** (**0.01; 2.11)**	0.83 (−0.27; 1.94)	0.74 (−0.30; 1.78)	0.71 (−0.32; 1.73)	0.50 (−0.51; 1.50)	0.51 (−0.43; 1.45)	0.22 (−1.39; 1.83)	CON

All results are presented in the form of SMD (95% CrI). various exercises are ranked according to the surface under the curve cumulative for overall effect starting with the best from left to right. The results of the network meta-analysis are showed in the lower left part, and results from pairwise comparisons in the upper right half (if available). Cells shown in bold indicate signifcant results. CON “control group”; breathing “diaphragm breathing exercsie”; papworth “Papworth breathing exercise”; IMT “inspiration muscle training”; buteyko “Buteyko breathing exercise”; aerobic “moderate-intensity aerobic exercise”; HIIT “High-intensity interval training”; pranayama “Pranayama exercise”; aerobic_breathing “aerobic combined with breathing exercise”; aerobic_resistance “aerobic combined with resistance exercise”.

**Figure 5:**
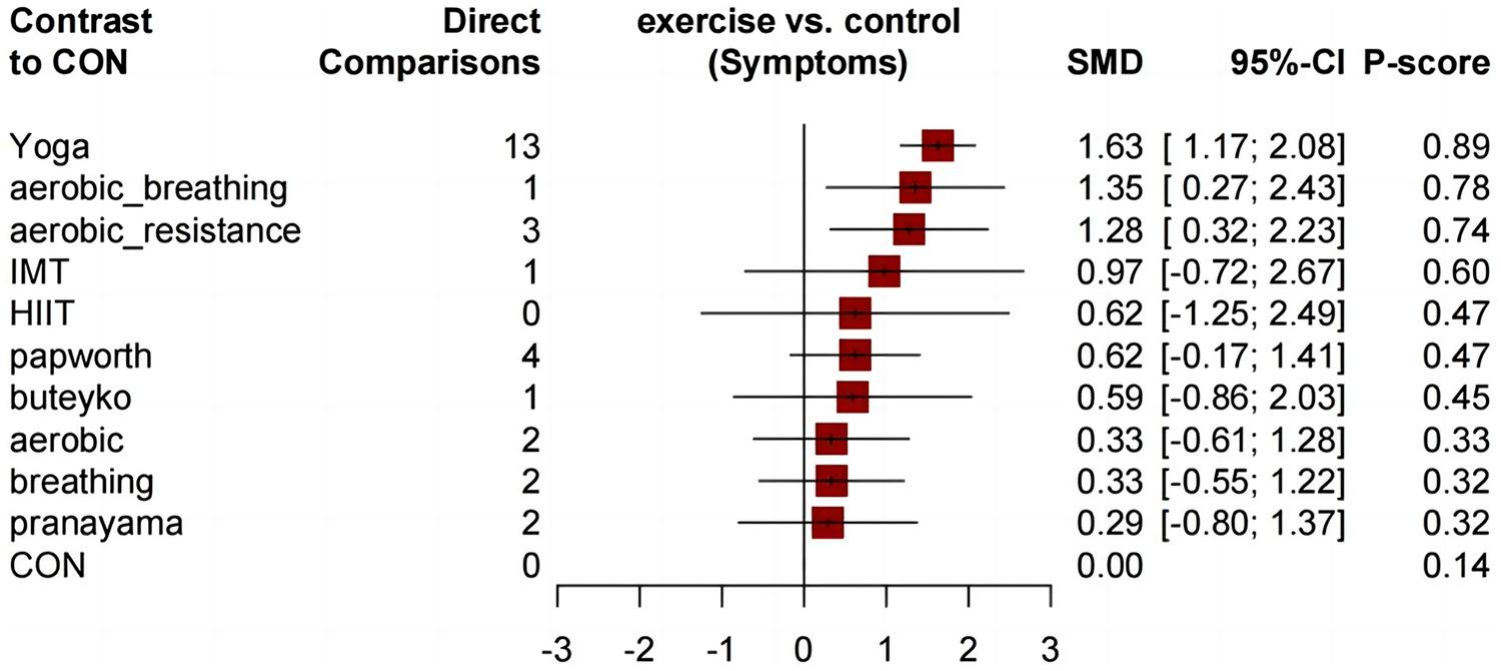
Forest plot change in effect of asthma QOL symptoms score.

**Figure 6:**
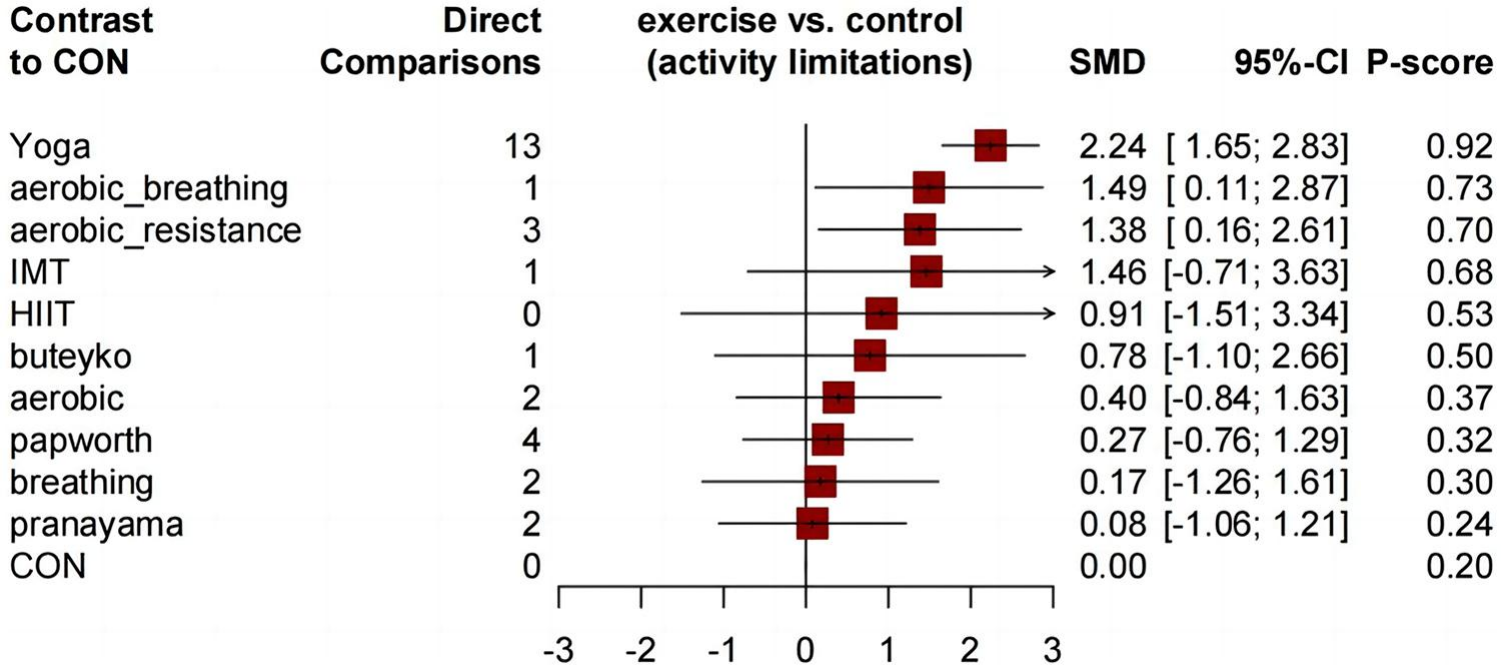
Forest plot change in effect of asthma QOL activity limitations score.

**Figure 7:**
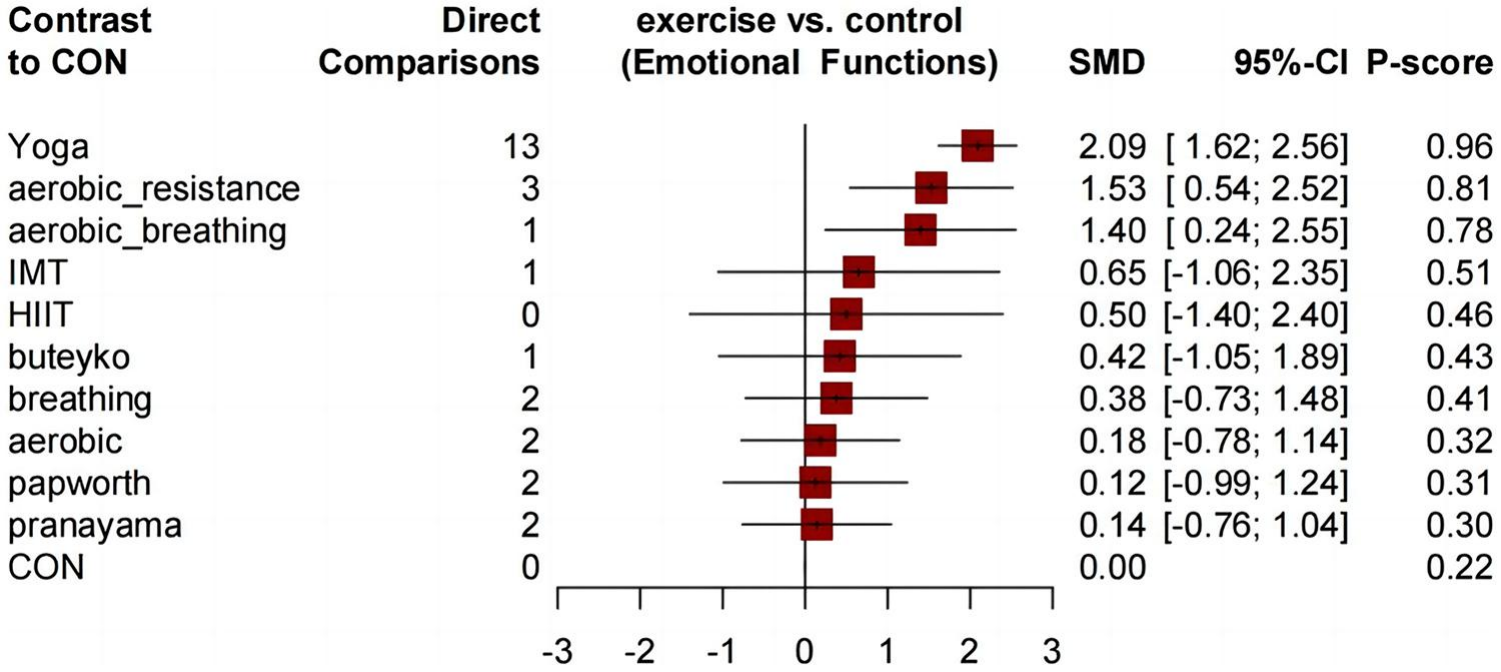
Forest plot change in effect of asthma QOL emotional functions score.

**Figure 8:**
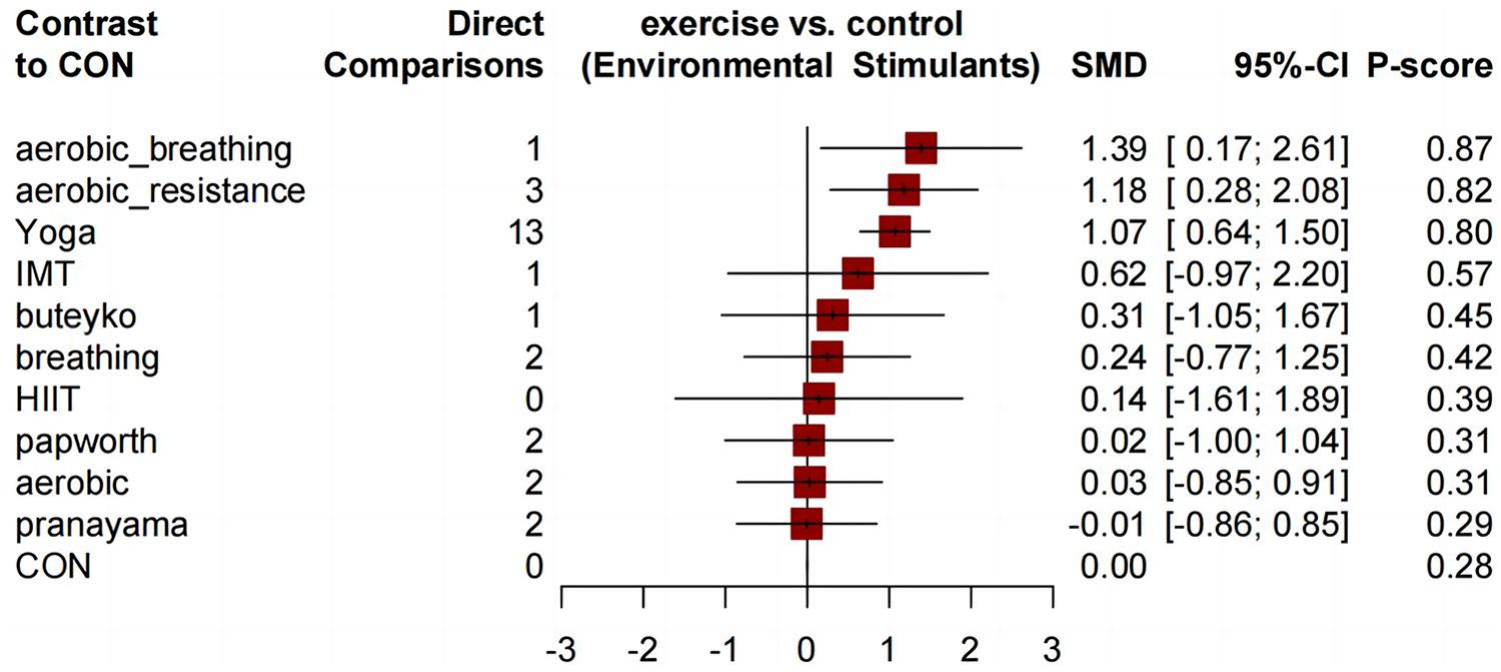
Forest plot change in effect of asthma QOL environmental stimulus score.

Similar to the asthma-related QOL total score, compared to the control group, all comprehensive exercises can significantly improve the QOL subdimension scores. Compared with the control group, all comprehensive-mode exercises significantly improved the asthma QOL symptoms score (Figure 5), and the SMDs [95% Credible Interval (CrI)] ranged between 1.63 (1.17 to 2.08) for Yoga to 1.28 (0.32 to 2.23) for aerobic_resistance, and Yoga ranks first (*P*-score = 0.89); for the asthma QOL activity limitations score, three comprehensive-mode exercises all show significant improvement (Figure 6), and the SMDs [95% Credible Interval (CrI)] ranged between 2.24 (1.65 to 2.83) for Yoga to 1.38 (0.16 to 2.61) for aerobic_resistance, and Yoga ranks first (*P*-score = 0.92); for the asthma QOL Emotional Functions score, three comprehensive-mode exercises all show significant improvement (Figure 7), and the SMDs [95% Credible Interval (CrI)] ranged between 2.09 (1.62 to 2.56) for Yoga to 1.40 (0.24 to 2.55) for aerobic_breathing, and Yoga ranks first (*P*-score = 0.96); for the asthma QOL Emotional Functions score, three comprehensive-mode exercises all show significant improvement (Figure 8), and the SMDs [95% Credible Interval (CrI)] ranged between 1.39 (0.17 to 2.61) for aerobic_breathing to 1.07 (0.64 to 1.50) for Yoga, and Yoga ranks first (*P*-score = 0.87). League table for changes in each subdimension QOL score associated with various exercise modes as showed in Supplementary Information S5.

The heterogeneity of most outcomes was high (Supplementary Information S9). None of the outcomes was significantly inconsistent according to the design-by-treatment interaction test (Supplementary Information S9). No small study effect was found for the primary outcome (Supplementary Information S10). Potential threats to the source of heterogeneity from baseline characteristics and exercise training doses of the included studies were resolved by meta-regression analysis of the primary outcome. Exercise periods, mean age and time of single session were significant factors affecting the network meta-analysis results (Supplementary Information S11).

## Discussion

### Overview

After conducting a detailed classification of exercise programs, pooled data indicates that the three types of integrated exercise programs are the most effective concerning the improvement of asthma QOL total score and subdimension scores. The application of solely moderate-intensity aerobic exercise shows insignificant enhancement in asthma-related QOL, which is far less compared to HIIT. Despite various breathing exercises that have a trend of improving asthma-related QOL, the differences in their effectiveness remain insignificant. IMT ranked the lowest, nearly ineffective. The assessment of asthma-related QOL, which involves a self-evaluation indicator, correlates with multiple factors encompassing physical health, psychological emotions, asthma symptoms, and clinical conditions (39). Hence, the effects of different exercise modes on asthma QOL are multi-dimensional.

### Single-mode exercise

#### Breathing exercise

Despite the results indicating that various breathing exercises merely demonstrate an improvement trend in QOL, the well-acknowledged benefits of ventilation techniques cannot be ignored. Particularly, during asthma exacerbation, inflammation and obstruction of airways compel patients to utilize additional upper chest respiratory muscles and resort to limited, rapid, shallow mouth breathing (40). Repeated breathing exercises can foster a more efficient breathing pattern, alleviating this respiratory dysfunction (41). Furthermore, asthmatics usually exhibit a lower End-tidal carbon dioxide (EtCO_2_) level (42), which will specifically cause an increase in airway resistance (hyperventilation syndrome). Various breathing techniques have been validated to elevate the EtCO_2_ level in asthmatics by modifying respiratory rate and ventilation volume to ameliorate symptoms and decrease reliance on medication (43). Another major benefit of breathing exercises for asthma is to alleviate asthma emotional disturbance (44), a risk factor affecting asthma incidence independently of objective indicators (45). Besides reducing the respiratory rate, traditional diaphragmatic breathing methods limit the involvement of auxiliary respiratory muscles and emphasize enhancing inhalation capacity under the overload principle to mitigate air hunger (46). Moreover, after regular diaphragmatic breathing exercises, the size and kinematics of the abdomen and chest of asthmatics can be significantly improved, which helps to improve lung function (47). Although the Papworth method enriches the diaphragmatic techniques with relaxation exercises (48), more extensive evidence is needed to ascertain the added value of these elements for providing additional benefits. Pranayama, although simple, chiefly seeks to decelerate and extend breathing. As an integral part of yoga, pranayama harmonizes physical and mental health by modulating the endocrine and sympathetic nervous system. A three-arm study illustrates that Buteyk breathing exercises can enhance the standard of living and asthma control more effectively than pranayama (18). The efficacy of Buteyko can be credited to its distinctive design, involving prolonged breath-holding combined with physical activities (49), thereby augmenting EtCO_2_ levels and contributing to the reduction of airway hyper-responsiveness. Furthermore, guidelines promoting nasal breathing and decreased medication usage foster asthma stability (50). Although any single form of breathing exercises may only partially yield the desired outcomes for asthma patients, their integration with physical training is a more effective way. Moreover, it is imperative to develop new ventilation techniques, capitalizing on the strengths of diverse ventilation technology.

#### Inspiratory muscle training (IMT)

IMT is an intervention aimed at enhancing inspiratory muscle function through the application of specific resistive loads (51). This meta-analysis revealed that IMT failed to demonstrate statistically significant enhancements in the overall QOL score (52). The current synthesis was limited to two controlled trials, with only one study reporting a statistically significant between-group difference favoring the IMT intervention (53). However, when evaluated via the minimally important difference (MID) as a clinical relevance metric, the other study demonstrated that the IMT group attained clinically meaningful improvements (≥0.5 points) in both global and domain-specific QOL scores, while the control group failed to reach this threshold (54). This implies that although the inter-group mean difference was statistically non-significant, IMT recipients may perceive a substantive enhancement in QOL, underscoring a divergence between statistical significance and clinical relevance. In additions, the analysis of the subdomain QOL score showed that the control group reached the MID only in emotional and environmental domains, with no significant improvement in symptom or activity domains. This suggests that IMT elicits domain-specific therapeutic effects (54). Current evidence indicates that IMT can improve inspiratory muscle strength in asthma patients, as measured by maximal inspiratory pressure (PImax) (53–57). This enhancement in PImax leading to a reduction in dyspnea and asthma symptoms, as stronger inspiratory muscles can work at a lower relative intensity, thereby reducing respiratory drive (58, 59). The physiological mechanism is based on the principle of respiratory muscle-specific training: IMT increases the load on respiratory muscles, promotes muscle fiber recruitment and metabolic adaptations, reduces respiratory effort, optimizes the force-length relationship, and thereby alleviates dynamic pulmonary hyperinflation (60). However, the improvement in inspiratory muscle strength depends on the training load relative to the initial PImax (52, 54). Additionally, IMT can improves inspiratory muscle endurance, allowing patients to breathe more efficiently for longer periods under resistance. The strengthening of inspiratory muscles directly contributes to improved endurance, as stronger muscles can operate at lower intensities and exhibit greater fatigue resistance (55). By enhancing both inspiratory muscle strength and endurance, IMT helps improve exercise tolerance and reduce the sensation of dyspnea. However, the effect of IMT on ventilatory function (e.g., FEV1, FVC) and exercise capacity remains controversial (52, 58). In conclusion, while IMT yields specific benefits for asthma patients, evidence regarding its impact on QOL is still insufficient and requires further investigation. The findings of this study suggest that future interventions should explore combining IMT with whole-body exercise to achieve more comprehensive functional improvements.

#### Whole-body exercise

The results indicate that conducting moderate-intensity aerobic exercise alone cannot lead to a significant enhancement in the asthma QOL. However, various benefits of aerobic exercise for asthma have been documented extensively (61). Notably, aerobic exercise, unlike respiratory exercises, can substantially upgrade the cardiopulmonary function and health functionality of asthma patients (62). Comprehensive aerobic exercise can intensify the residual airflow for these patients (63), besides fortifying bronchial expansion (64), resulting in improved ventilation. This adaptivity can mitigate the limitations of asthmatics activity. Furthermore, comprehensive aerobic activity may aid in reducing sensitivity to accumulated fear of respiratory distress in asthma patients (65), thereby elevating their activity limitations. An RCT indicates that a higher percentage of aerobic exercise participants witness more sustained improvements in both asthma management and medication application (66) than respiratory exercise participants, all contributing to enhanced QOL. Asthma is conceptualized as an inflammatory process, with chronic airway inflammation instigated by cytokines and other inflammatory mediators (67). Numerous studies utilizing mouse models have shown that repeated moderate-intensity aerobic exercises can substantially alleviate systemic and airway inflammation (68, 69). The regulation of Th1/Th2 balance is crucial in the immunotherapy of asthma (70). Moreover, multiple studies have shown a correlation between the systemic concentration of proinflammatory cytokines like IL-4 and IFN-*γ* and aerobic exercise in asthmatics (71, 72). After regular aerobic exercise, there is a trend of reduced levels of inflammatory mediators like eosinophil cationic protein (ECP) of asthmatics (73). However, it is imperative to acknowledge ongoing debates regarding the effectiveness of aerobic exercise in anti-inflammatory effect on asthmatics (74), partly attributing to the diverse methodologies, such as exercise prescriptions, participant characteristics, and medication management.

Compared with low to medium-intensity training, HIIT is frequently employed as a therapy program for cardiovascular diseases, owing to its superior effects on augmenting cardiorespiratory health and endurance performance (75). The results illustrated that HIIT had a superior impact compared to moderate-intensity aerobic exercises; however, most rehabilitation guidelines recommend asthma patients to engage in more moderate-intensity whole-body exercises (2, 76). Moreover, high-intensity anaerobic exercise is more likely to induce EIB (77). Nonetheless, only a limited number of studies have confirmed the viability of HIIT in treating asthma (involving moderate or severe persistent, obese or overweight, postmenopausal women, and elderly asthma patients) (78, 79). Silva et al. indicated that HIIT can improve fatigue, symptoms of breathing difficulty, and activity limitations in asthmatic patients compared to moderate-intensity sustained aerobic exercise (17). This amelioration is associated with the escalated rate of lactic acid clearance after regular HIIT (80) and relief of central fatigue stimulation (81). Although there is no direct evidence in asthmatic patients, compared to moderate-intensity aerobic exercise, HIIT can enhance VO_2_ max more in healthy individuals or athletes (82). Among asthmatics, an increase in VO_2_ max usually coincides with a raised threshold of respiratory discomfort, enabling them to manage everyday life activities with less effort and yielding surplus respiratory reserves. Nevertheless, it is imperative to note that there is a lack of research specifically focused on administering HIIT as a pulmonary revival program for influencing the clinical outcomes of asthma; thereby, further studies are required concerning the broad spectrum of asthma phenotypes.

### Comprehensive-mode exercise

#### Aerobic combining breathing or resistance exercise

Contrary to previous studies, this study has defined the exercise program more explicitly. The findings reveal that comprehensive exercise programs combining aerobic and strength or breathing exercises can notably ameliorate the QOL for individuals with asthma. Shaw et al. showed that people with asthma experience significant improvements in lung function, abdominal and thoracic dimensions, and kinematics when participating in the breathing, aerobic, and combined groups as opposed to the non-exercise group (83). The efficacy of combined courses is significantly better than single-mode exercise, and there will be a synergistic effect rather than interference between aerobic exercise and respiratory exercise. Despite strength training rarely being utilized individually within asthma rehabilitation programs, no study has revealed the unique role of this exercise element. Nonetheless, national health organizations advocate incorporating strength training into comprehensive fitness programs, including aerobic and flexibility exercises, due to their extensive benefits to health and performance (84). Metabolic traits (85), cardiovascular functionality, and muscle and connective tissue cross-sectional areas (86) reveal the adaptive changes following regular strength training, which may enhance mobility in asthma patients. Furthermore, integrating aerobic exercises with strength training is particularly efficient at reducing body fat (87). For overweight and obese adult asthma patients, a moderate weight reduction (5%–10% of total body weight) can significantly improve lung functionality, asthma management, and overall QOL (88, 89). Moreover, a reduction in fat or BMI may influence systemic and airway inflammation, potentially reducing the frequency of asthma symptoms (90).

#### Yoga

The onset of asthma closely correlates with patients' emotional distress and physiological obstacles (91), which directly affects the QOL. Pooled data indicate that yoga ranks the highest among all methods in both the overall QOL and sub-dimension scores. The benefits of yoga to health and various diseases have been thoroughly demonstrated since its first systematic application to medicine (92). The comprehensive technical system of yoga (posture, breathing, and mind) can promote the overall development of individuals in a balanced manner by enhancing the mind-body connection (93). It is known that asthma patients often feel fear, stress, and anxiety. The biopsychosocial theory posits that this psychological disturbance is closely related to individuals' health status, which is not only the result of an asthma attack but also its cause. Yoga can help asthmatics achieve a “relaxed state” to alleviate negative emotions. Researchers showed that yoga can improve the balance of autonomous nervous system activity (94) in asthmatics and lower levels of saliva cortisol. Meditation can further shift individual mindset by boosting confidence and motivation (95), augmenting cognitive function and activity levels. Vempati et al. postulated that the reduction in mast cell activation levels in asthmatic individuals could be attributed to the positive effects of yoga on emotional well-being, leading to a decrease in inflammation and symptoms (96). This occurs because, apart from the activation of mast cells by immunoglobulin E (IgE), emotional stress triggers the secretion of dura mater mast cells by releasing adrenocorticotropic hormone-releasing hormones (97). Furthermore, acute psychological stress can induce heart mast cell degranulation either directly via CRH or neurotensin (98). Moreover, correcting the disordered breathing pattern in asthmatics via breath regulation is feasible, whereas physical fitness can be escalated through posture practice, potentially minimizing asthma activity limitations (99). While the benefits of yoga are multifaceted, further research is required to confirm the specific mechanisms targeting asthma (such as inflammatory cells, methacholine, and Urinary 11*β* prostaglandin F2*α*) (100).

Notably, the completeness of the yoga program has been a key point of controversy in the past about the therapeutic effects of yoga (13). Some studies conveniently used the blind method on the control group by substituting regular stretching or relaxation exercises for postures or excluding meditation (101, 102). However, this approach was found to diminish the efficacy of yoga. Although the meta-analysis by Cramer et al. suggests that yoga improves QOL, the lack of direct comparisons has prevented the investigation of the differences between various elements of yoga (13). The findings from the combinational analysis involving direct and indirect comparisons suggest that a comprehensive yoga program greatly outperforms pranayama alone in improving the QOL for asthmatics. Each element in this mind-body practice has its unique function. Only the combined use of these elements may optimize the therapeutic effect.

#### Exercise prescription variables

In addition to the diversification of exercise regimens, careful consideration must be given to the configuration of exercise prescription variables. Meta-regression of the primary outcome assessed potential heterogeneity arising from training doses (including exercise duration, frequency, and session time). The hierarchy observed in the unadjusted model remained consistent following adjustment for the centering values of all covariates. Nonetheless, the intervention period was identified as a significant effect modifier. When the model was adjusted for the centering value of the intervention period (10.0456 weeks), the effects of various breathing techniques demonstrated statistical significance compared with the control group. These findings suggest that longer intervention periods are required to achieve more substantial improvements in quality of life. For a chronic condition such as asthma, long-term disease control is dependent upon sustained adherence to rehabilitation guidelines (103, 104). The integration of physical activity into daily life as a habitual practice is essential for its effectiveness as a foundational non-pharmacological management strategy. Furthermore, several included studies implemented a frequent, accumulated breathing exercise regimen, characterized by multiple short sessions of breathing training distributed throughout the day (43, 102, 105, 106). For the general population, such an accumulated exercise schedule has been shown to support health maintenance and improve physical fitness (107, 108), while simultaneously accommodating the demands of modern lifestyles. However, there is currently a lack of empirical research exploring potential differences in effects on asthma control or quality of life between a consolidated (a single prolonged session on certain days of the week) and a distributed (high-frequency accumulated) breathing exercise program in asthma patients. The effectiveness of such regimens is likely to be associated with asthma severity, highlighting the need for personalized exercise prescriptions.

### Limitations

This study investigates the therapeutic impacts of various exercise modes on asthma-related QOL in patients. However, it is difficult to quantitatively evaluate supplementary components within the treatment regimen, such as integrated management models, non-exercise rehabilitation approaches, the development of exercise prescriptions and condition. Professional volunteers provide face-to-face medical supervision, ensuring the efficacy of rehabilitation programs. Nevertheless, this approach is more challenging for most patients due to logistical and financial challenges, as well as insufficient access to professional physical therapists. Previous research indicates that the efficacy of DVD-based (109) or home exercises (110, 111) with regular follow-ups or tele-reminders is comparable to face-to-face physiotherapy; however, the limited sample size and variability in participant engagement render it uncertain whether these methods consistently ensure adequate adherence and therapeutic effectiveness. Moreover, some studies lack any mention of supervision methods to ensure participant compliance—a cornerstone of effective asthma control.

It is known that additional non-pharmacological rehabilitation elements, such as asthma education and dietary therapy, are beneficial to the QOL of asthma patients. However, the efficacy of exercise rehabilitation may be weakened. The limited scope of available studies also constrains the feasibility of conducting subgroup analyses. Furthermore, the doctor-patient relationship indirectly influences the therapeutic effect of physical therapy, although it is difficult to distinguish. Difference in asthma severity and incidence among patients introduce significant heterogeneity, affecting the reliability of findings. Variation in movement capacity and asthma control across severity levels fundamentally limits the feasibility and safety of completing rehabilitation programs. However, many studies neglect these differences by applying uniform exercise regimens to patients across all severity levels, often with insufficient supervision. This approach adversely affects adherence and diminishes therapeutic effectiveness. Furthermore, although pre-intervention asthma education is commonly provided, some patients are still unable to complete the programs due to health-related issues, resulting in missing data or bias. Such limitations not only undermine the robustness of empirical findings but also affect the credibility of ranked models. Thus, there is a clear need to develop more systematic and individualized rehabilitation strategies that incorporate patient-specific factors—such as clinical characteristics, physical function, and socioeconomic status—while implementing stronger oversight to improve adherence.

## Conclusion

Compared to single-mode exercise, a comprehensive program can improve asthma-related QOL more effectively. Yoga, which incorporates physical activity, breathing techniques, and psychological regulation, has shown the best effect. Low-quality evidence suggests that HIIT may be more effective in improving asthma QOL than moderate-intensity aerobic training. The exercise period will affect the effectiveness of the non-pharmacological rehabilitation program.

## Data Availability

The original contributions presented in the study are included in the article/Supplementary Material, further inquiries can be directed to the corresponding author.
