# Supplementary material for: Comprehensive exercise program based on optimal physiotherapy for asthma-related quality of life: a systematic review and network meta-analysis
Source: Front Sports Act Living. 2026 Jan 20;7:1738390. doi: 10.3389/fspor.2025.1738390 (PMC12865712; doi:10.3389/fspor.2025.1738390)
Supplement: Supplementary file 1 [file Datasheet1.pdf]

Supplementary Information

Supplementary Information 1: PRISMA Checklist

Section/Topic	Item #	Checklist Item	Reported on Page #
**TITLE**			
Title	1	Comprehensive exercise program is the optimal physiotherapy for Asthma quality of life: a systematic review and network meta-analysis	Title
**ABSTRACT**			
Structured summary	2	Provide a structured summary including, as applicable:<br>**Background:** main objectives<br>**Methods:** data sources; study eligibility criteria, participants, and interventions; study appraisal; and *synthesis methods, such as network meta-analysis.*<br>**Results:** number of studies and participants identified; summary estimates with corresponding confidence/credible intervals; *treatment rankings may also be discussed. Authors may choose to summarize pairwise comparisons against a chosen treatment included in their analyses for brevity.*<br>**Discussion/Conclusions:** limitations; conclusions and implications of findings.<br>**Other:** systematic review registration number with registry name.	Abstract
**INTRODUCTION**			
Rationale	3	Describe the rationale for the review in the context of what is already known*, including mention of why a network meta-analysis has been conducted.*_	Introduction
Objectives	4	Provide an explicit statement of questions being addressed, with reference to participants, interventions, comparisons, outcomes, and study design (PICOS).	Introduction
**METHODS**			
Protocol and registration	5	Indicate whether a review protocol exists and if and where it can be accessed (e.g., Web address); and, if available, provide registration information, including registration number.	Methods, PROSPERO (CRD42023421683)
Eligibility criteria	6	Specify study characteristics (e.g., PICOS, length of follow-up) and report characteristics (e.g., years considered, publication status) used as criteria for eligibility, giving rationale. *Clearly describe eligible treatments included in the treatment network, and note whether any have been clustered or merged into the same node (with justification).*_	Study selection

Information sources	7	Describe all information sources (e.g., databases with dates of coverage, contact with study authors to identify additional studies) in the search and date last searched.	Data source and search
Search	8	Present full electronic search strategy for at least one database, including any limits used, such that it could be repeated.	Supplementary Information3
Study selection	9	State the process for selecting studies (i.e., screening, eligibility, included in systematic review, and, if applicable, included in the meta-analysis).	Study selection
Data collection process	10	Describe method of data extraction from reports (e.g., piloted forms, independently, in duplicate) and any processes for obtaining and confirming data from investigators.	Data Abstraction And Quality assessment
Data items	11	List and define all variables for which data were sought (e.g., PICOS, funding sources) and any assumptions and simplifications made.	
**Geometry of the network**	**S1**	Describe methods used to explore the geometry of the treatment network under study and potential biases related to it. This should include how the evidence base has been graphically summarized for presentation, and what characteristics were compiled and used to describe the evidence base to readers.	Statistical analysis
Risk of bias within individual studies	12	Describe methods used for assessing risk of bias of individual studies (including specification of whether this was done at the study or outcome level), and how this information is to be used in any data synthesis.	Data Abstraction And Quality assessment
Summary measures	13	State the principal summary measures (e.g., risk ratio, difference in means).	Statistical Analysis, Supplementary Information 6
Planned methods of analysis	14	Describe the methods of handling data and combining results of studies for each network meta-analysis. This should include, but not be limited to:<br>• *Handling of multi-arm trials;*<br>• *Selection of variance structure;*<br>• *Selection of prior distributions in Bayesian analyses; and*<br>• *Assessment of model fit.*	Statistical Analysis, Supplementary Information6
**Assessment of Inconsistency**	**S2**	Describe the statistical methods used to evaluate the agreement of direct and indirect evidence in the treatment network(s) studied. Describe efforts taken to address its presence when found.	Statistical Analysis, Supplementary Information 6 and 8
Risk of bias across studies	15	Specify any assessment of risk of bias that may affect the cumulative evidence (e.g., publication bias, selective reporting within studies).	Supplementary Information 10

Additional analyses	16	Describe methods of additional analyses if done, indicating which were pre-specified. This may include, but not be limited to, the following:<br>• Sensitivity or subgroup analyses;<br>• Meta-regression analyses;<br>• *Alternative formulations of the treatment network; and*<br>• *Use of alternative prior distributions for Bayesian analyses (if applicable).*_	Supplementary Information 11
**RESULTS†**			
Study selection	17	Give numbers of studies screened, assessed for eligibility, and included in the review, with reasons for exclusions at each stage, ideally with a flow diagram.	Study characteristics and quality assessment, Figure 1
**Presentation of network structure**	**S3**	Provide a network graph of the included studies to enable visualization of the geometry of the treatment network.	Study characteristics and quality assessment, Figure 2; Supplementary Information 5
**Summary of network geometry**	**S4**	Provide a brief overview of characteristics of the treatment network. This may include commentary on the abundance of trials and randomized patients for the different interventions and pairwise comparisons in the network, gaps of evidence in the treatment network, and potential biases reflected by the network structure.	Study characteristics and quality assessment, Figure 2; Supplementary Information 5
Study characteristics	18	For each study, present characteristics for which data were extracted (e.g., study size, PICOS, follow-up period) and provide the citations.	Table 1,
Risk of bias within studies	19	Present data on risk of bias of each study and, if available, any outcome level assessment.	Supplementary Information 10
Results of individual studies	20	For all outcomes considered (benefits or harms), present, for each study: 1) simple summary data for each intervention group, and 2) effect estimates and confidence intervals. *Modified approaches may be needed to deal with information from larger networks.*	
Synthesis of results	21	Present results of each meta-analysis done, including confidence/credible intervals. *In larger networks, authors may focus on comparisons versus a particular comparator (e.g. placebo or standard care), with full findings presented in an appendix. League tables and forest plots may be considered to summarize pairwise comparisons.* If additional summary	Figure 3 and Table 2, and Supplementary Information 5

		measures were explored (such as treatment rankings), these should also be presented.	
**Exploration for inconsistency**	**S5**	Describe results from investigations of inconsistency. This may include such information as measures of model fit to compare consistency and inconsistency models, *P* values from statistical tests, or summary of inconsistency estimates from different parts of the treatment network.	Supplementary Information 9
Risk of bias across studies	22	Present results of any assessment of risk of bias across studies for the evidence base being studied.	Network meta analysis, Supplementary Information 9
Results of additional analyses	23	Give results of additional analyses, if done (e.g., sensitivity or subgroup analyses, meta-regression analyses*, alternative network geometries studied, alternative choice of prior distributions for Bayesian analyses,* and so forth).	Network meta analysis, Supplementary Information 11
**DISCUSSION**			
Summary of evidence	24	Summarize the main findings, including the strength of evidence for each main outcome; consider their relevance to key groups (e.g., healthcare providers, users, and policy-makers).	Overview
Limitations	25	Discuss limitations at study and outcome level (e.g., risk of bias), and at review level (e.g., incomplete retrieval of identified research, reporting bias). *Comment on the validity of the assumptions, such as transitivity and consistency. Comment on any concerns regarding network geometry (e.g., avoidance of certain comparisons).*	Limitations
Conclusions	26	Provide a general interpretation of the results in the context of other evidence, and implications for future research.	Conclusions
**FUNDING**			
Funding	27	Describe sources of funding for the systematic review and other support (e.g., supply of data); role of funders for the systematic review. This should also include information regarding whether funding has been received from manufacturers of treatments in the network and/or whether some of the authors are content experts with professional conflicts of interest that could affect use of treatments in the network.	21

PICOS = population, intervention, comparators, outcomes, study design.

Supplementary Information 2: Protocol

Review title and timescale

1. Review title.

Give the title of the review in English

Comprehensive exercise program is the optimal physiotherapy for Asthma quality of life: a systematic review and network meta-analysis

2. Original language title.

For reviews in languages other than English, give the title in the original language. This will be displayed with the English language title.

3. Anticipated or actual start date.

Give the date the systematic review started or is expected to start.

1/11/2024

4. Anticipated completion date.

Give the date by which the review is expected to be completed.

31/3/2024

5. Stage of review at time of this submission.

Tick the boxes to show which review tasks have been started and which have been completed. Update this field each time any amendments are made to a published record.

Reviews that have started data extraction (at the time of initial submission) are not eligible for inclusion in PROSPERO. If there is later evidence that incorrect status and/or completion date has been supplied, the published PROSPERO record will be marked as retracted.

This field uses answers to initial screening questions. It cannot be edited until after registration.

The review has not yet started:

Review stage	Started	Completed
Preliminary searches	Yes	No
Piloting of the study selection process	Yes	No
Formal screening of search results against eligibility criteria	No	No
Data extraction	No	No
Risk of bias (quality) assessment	No	No
Data analysis	No	No

Provide any other relevant information about the stage of the review here.

6. Named contact.

The named contact is the guarantor for the accuracy of the information in the register record. This may be any member of the review team.

Xinmiao Feng

Email salutation (e.g. "Dr Smith" or "Joanne") for correspondence:

Dr Feng

7. Named contact email.

Give the electronic email address of the named contact.

fengxinmiao666@163.com

8. Named contact address

Give the full institutional/organisational postal address for the named contact.

Xinxi Street, Haidian District, Beijing, China

9. Named contact phone number.

Give the telephone number for the named contact, including international dialling code.

19933227661

10. Organisational affiliation of the review.

Full title of the organisational affiliations for this review and website address if available. This field may be completed as 'None' if the review is not affiliated to any organisation.

Sports coaching college, Beijing Sport University, 100084, Haidian District, Beijing, China

Organisation web address:

11. Review team members and their organisational affiliations.

Give the personal details and the organisational affiliations of each member of the review team. Affiliation refers to groups or organisations to which review team members belong. **NOTE: email and country now MUST be entered for each person, unless you are amending a published record.**

Dr Xinmiao Feng. Sports coaching college, Beijing Sport University, 100084, Beijing, China

Dr Yonghui Chen. Sports coaching college, Beijing Sport University, 100084, Beijing, China

Dr Peide Cao. Hainan Normal University, 571158, Haikou, China

12. Funding sources/sponsors.

Details of the individuals, organizations, groups, companies or other legal entities who have funded or sponsored the review.

None

13. Conflicts of interest.

List actual or perceived conflicts of interest (financial or academic).

None

14. Collaborators.

Give the name and affiliation of any individuals or organisations who are working on the review but who are not listed as review team members. NOTE: email and country must be completed for each person, unless you are amending a published record.

15. Review question.

State the review question(s) clearly and precisely. It may be appropriate to break very broad questions down into a series of related more specific questions. Questions may be framed or refined using PI(E)COS or similar where relevant.

1) Are exercise interventions more effective than non-exercise interventions for quality of life in adults with asthma?

2) Are there specific physical activities or exercise interventions that are more effective than others for quality of life in adults with asthma?

16. Searches.

State the sources that will be searched (e.g. Medline). Give the search dates, and any restrictions (e.g. language or publication date). Do NOT enter the full search strategy (it may be provided as a link or attachment below.)

The systematic review and meta-analysis were conducted in accordance with the Preferred Reporting Items for Systematic Reviews and Meta-Analyses (PRISMA) guidelines. We will search PubMed, EMBASE, The Cochrane Library (Cochrane Database of Systematic Reviews, and Web of Science. Studies published between inception and the date the searches are run will be sought. The searches will be re-run just before the final analyses and further studies retrieved for inclusion. We will use a Boolean search strategy with the operators AND, OR, NOT, and the search strategy will include terms describing or relating to intervention, participants, and study design.

17. URL to search strategy.

Upload a file with your search strategy, or an example of a search strategy for a specific database, (including the keywords) in pdf or word format. In doing so you are consenting to the file being made publicly accessible. Or provide a URL or link to the strategy. Do NOT provide links to your search results.

I give permission for this file to be made publicly available

Yes

18. Condition or domain being studied.

Give a short description of the disease, condition or healthcare domain being studied in your systematic review.

Asthma and exercise

19. Participants/population.

Specify the participants or populations being studied in the review. The preferred format includes details of both inclusion and exclusion criteria.

Age: 'adult' ('16+') . Sex: Males and females

Race: Any

Diagnosed Asthma

20. Intervention(s), exposure(s).

Give full and clear descriptions or definitions of the interventions or the exposures to be reviewed. The preferred format includes details of both inclusion and exclusion criteria.

The intervention to be reviewed is: I. Exercise based intervention and II. Types of exercise intervention in multi-treatment comparison, including

1) yoga

2) breathing exercise (diaphragm)

3) Papworth breathing exercise

4) Pranayama breathing exercise

5) Buteyko breathing exercise

6) inspiratory muscles training

7) moderate-intensity aerobic exercise

8) Interval high-intensity training

9) aerobic exercise combined with breathing exercise

10) aerobic exercise combined with strength training

21. Comparator(s)/control.

Where relevant, give details of the alternatives against which the intervention/exposure will be compared (e.g. another intervention or a non-exposed control group). The preferred format includes details of both inclusion and exclusion criteria.

1. 'Usual care' control was determined based on the report. In 'usual care', participants were expected to continue the routine standard of care provided by their general practitioners.

2. Control groups that were not given any specific intervention such as 'waiting list' or usual physical activity or asthma conduction or where the authors did not specify the nature of the control were also classified as 'usual care'. 'Waiting-list' controls were given active intervention after a period of observation, with no new intervention being delivered during the trial period.

22. Types of study to be included.

Give details of the study designs (e.g. RCT) that are eligible for inclusion in the review. The preferred format includes both inclusion and exclusion criteria. If there are no restrictions on the types of study, this should be stated.

Include only randomized controlled trials

23. Context.

Give summary details of the setting or other relevant characteristics, which help define the inclusion or exclusion criteria.

24. Main outcome(s).

Give the pre-specified main (most important) outcomes of the review, including details of how the outcome is

Total score and subdimension score of the asthma QOL questionnaire (the asthma QOL questionnaire; Mini AQLQ; St George’s Respiratory Questionnaire)

Measures of effect

Please specify the effect measure(s) for you main outcome(s) e.g. relative risks, odds ratios, risk difference, and/or 'number needed to treat.

Timing: The specified end point of the trial will be used as the end-point in the assessment.

Effect measure: Standardised mean difference at the end of the study

25. Additional outcome(s).

List the pre-specified additional outcomes of the review, with a similar level of detail to that required for main outcomes. Where there are no additional outcomes please state ‘None’ or ‘Not applicable’ as appropriate to the review

None

Measures of effect

Please specify the effect measure(s) for you additional outcome(s) e.g. relative risks, odds ratios, risk difference, and/or 'number needed to treat.

26. Data extraction (selection and coding).

Describe how studies will be selected for inclusion. State what data will be extracted or obtained. State how this will be done and recorded.

All search results will be exported into EndNote and duplicates will be removed. Titles and abstracts from the initial literature search will be independently assessed by two reviewers (X.M.F and L.L.Z). Full texts for articles deemed eligible for inclusion from the title and abstract search by either reviewer, in addition to those where no decision could be reached by the reviewers from this initial screen, will be screened independently by two researchers (X.M.F and L.L.Z). Any discrepancies will be resolved by discussion with all researchers in the review team. Two reviewers (X.M.F and L.L.Z) will independently extract data from the final inclusion list of articles into a standardised data extraction spreadsheet in Excel. At this stage, two authors extracted information on (1) relevant data regarding participant characteristics (e.g., the sample size, age, and sex); (2) training mode; (3) training variable (e.g., duration, frequency, sets, repetitions, and intensity) and (4) main result of the study. In case of incomplete raw data availability, we contacted the corresponding author of the manuscript. We excluded the studies of which the authors could not be reached. All studies were assessed independently in this systematic review by two researchers (X.M.F and L.L.Z) based on the extracted information. If there were any disagreements about the inclusion of a study, a third reviewer (Y.H.C) was consulted.

27. Risk of bias (quality) assessment.

State which characteristics of the studies will be assessed and/or any formal risk of bias/quality assessment tools that will be used.

The study quality was assessed with the PEDro scale, based on the list of Delphi [1]. The PEDro scale includes 11 items with three items from the Jadad scale [2] and nine items from the Delphi list [1]. PEDro rates RCTs on a scale from 0 (low quality) to 10 (high quality), and scores less than 6 are considered to have low methodological quality, as per the PEDro database statistics [3]. Interrater reliability was shown to be fair to good (Intraclass Correlation Coefficient = 0.68). Two reviewers (X.M.F and L.L.Z) scored the studies according to the proposed scale. In case of disagreements, a consensus was adopted or, if necessary, a third reviewer evaluated the article (Y.H.C).

28. Strategy for data synthesis.

Describe the methods you plan to use to synthesise data. This must not be generic text but should be specific to your review and describe how the proposed approach will be applied to your data.

If meta-analysis is planned, describe the models to be used, methods to explore statistical heterogeneity, and software package to be used.

Aggregated data will be used and a narrative synthesis will be presented. In addition, a quantitative synthesis is planned. Network meta-analysis is planned for the multiple treatment comparison. Heterogeneity will be assessed, in addition to consistency which will be examined by fitting consistency and inconsistency models. This will determine if the treatment effect for a pair of treatments estimated from an indirect comparison is consistent with the treatment effect estimated from a direct comparison.

29. Analysis of subgroups or subsets.

State any planned investigation of ‘subgroups’. Be clear and specific about which type of study or participant will be included in each group or covariate investigated. State the planned analytic approach.

None

30. Type and method of review.

Select the type of review, review method and health area from the lists below.

Network meta-analysis

31. Language.

Select each language individually to add it to the list below, use the bin icon to remove any added in error.

English

32. Country.

Select the country in which the review is being carried out. For multi-national collaborations select all the countries involved.

China

33. Other registration details.

Name any other organisation where the systematic review title or protocol is registered (e.g. Campbell, or The Joanna Briggs Institute) together with any unique identification number assigned by them. If extracted data will be stored and made available through a repository such as the Systematic Review Data Repository (SRDR), details and a link should be included here. If none, leave blank.

34. Reference and/or URL for published protocol.

If the protocol for this review is published provide details (authors, title and journal details, preferably in Vancouver format)

I give permission for this file to be made publicly available

Yes

35 Dissemination plans

Give brief details of plans for communicating essential messages from the review to the appropriate audiences.

Do you intend to publish the review on completion?

Yes

36 Keywords

Give words or phrases that best describe the review. (One word per box, create a new box for each term)

37. Details of any existing review of the same topic by the same authors.

If you are registering an update of an existing review give details of the earlier versions and include a full bibliographic reference, if available.

38. Current review status.

Update review status when the review is completed and when it is published.

New registrations must be ongoing so this field is not editable for initial submission.

Ongoing

39. Any additional information.

Provide any other information relevant to the registration of this review.

This study is only part of a randomized controlled experiment. Through this study, we want to find the best physical activity type to improve patients with asthma, and use this as the experimental group of our randomized controlled trial, and compare different exercise doses to Asthma's patients.

40. Details of final report/publication(s) or preprints if available.

Leave empty until publication details are available OR you have a link to a preprint (NOTE: this field is not editable for initial submission).

List authors, title and journal details preferably in Vancouver format.

Give the link to the published review or preprint

1 Verhagen, A. P. *et al.* The Delphi list: a criteria list for quality assessment of randomized clinical trials for conducting systematic reviews developed by Delphi consensus. *J Clin Epidemiol* **51**, 1235-1241, doi:10.1016/s0895-4356(98)00131-0 (1998).
2 Jadad, A. R. *et al.* Assessing the quality of reports of randomized clinical trials: is blinding necessary? *Control Clin Trials* **17**, 1-12, doi:10.1016/0197-2456(95)00134-4 (1996).
3 Maher, C. G., Sherrington, C., Herbert, R. D., Moseley, A. M. & Elkins, M. Reliability of the PEDro scale for rating quality of randomized controlled trials. *Phys Ther* **83**, 713-721 (2003).
4 Plowman, S. A. & Smith, D. L. *Exercise physiology for health fitness and performance*. (Lippincott Williams & Wilkins, 2013).
5 Ross, L. M., Porter, R. R. & Durstine, J. L. High-intensity interval training (HIIT) for patients with chronic diseases. *Journal of sport and health science* **5**, 139-144 (2016).
6 Hamasaki, H. Effects of diaphragmatic breathing on health: a narrative review. *Medicines* **7**, 65 (2020).
7 Saraswati, S. N. *Prana and pranayama*. (Yoga Publication Trust, 2009).
8 Bruton, A. & Lewith, G. T. The Buteyko breathing technique for asthma: a review. *Complementary therapies in medicine* **13**, 41-46 (2005).
9 Holloway, E. A. & West, R. J. Integrated breathing and relaxation training (the Papworth method) for adults with asthma in primary care: a randomised controlled trial. *Thorax* **62**, 1039-1042 (2007).
10 Powell, K. E., Paluch, A. E. & Blair, S. N. Physical activity for health: What kind? How much? How intense? On top of what? *Annual review of public health* **32**, 349-365 (2011).
11 Silva, I. S. *et al.* Inspiratory muscle training for asthma. *Cochrane Database of Systematic Reviews* (2013).
12 Khalsa, S. Yoga as a therapeutic intervention. *Principles and practice of stress management* **3**, 449-462 (2007).
13 Bahçecioğlu Turan, G. & Tan, M. The effect of yoga on respiratory functions, symptom control and life quality of asthma patients: A randomized controlled study. *Complement Ther Clin Pract* **38**, 101070, doi:10.1016/j.ctcp.2019.101070 (2020).

14 Erdoğan Yüce, G. & Taşcı, S. Effect of pranayama breathing technique on asthma control, pulmonary function, and quality of life: A single-blind, randomized, controlled trial. *Complement Ther Clin Pract* **38**, 101081, doi:10.1016/j.ctcp.2019.101081 (2020).

15 Thomas, M. *et al.* Breathing exercises for asthma: a randomised controlled trial. *Thorax* **64**, 55-61, doi:10.1136/thx.2008.100867 (2009).

16 França-Pinto, A. *et al.* Aerobic training decreases bronchial hyperresponsiveness and systemic inflammation in patients with moderate or severe asthma: a randomised controlled trial. *Thorax* **70**, 732-739 (2015).

17 Vempati, R., Bijlani, R. & Deepak, K. K. The efficacy of a comprehensive lifestyle modification programme based on yoga in the management of bronchial asthma: A randomized controlled trial. *BMC Pulmonary Medicine* **9**, doi:10.1186/1471-2466-9-37 (2009).

18 Türk, Y. *et al.* Short-term and long-term effect of a high-intensity pulmonary rehabilitation programme in obese patients with asthma: a randomised controlled trial. *European Respiratory Journal* **56** (2020).

19 Ma, J. *et al.* Behavioral Weight Loss and Physical Activity Intervention in Obese Adults with Asthma A Randomized Trial. *Annals of the American Thoracic Society* **12**, 1-11, doi:10.1513/AnnalsATS.201406-271OC (2015).

20 M, M., K, M., M, B. & B, H. Effect of 6 months of yoga practice on quality of life among patients with asthma: A randomized control trial. *Advances in Integrative Medicine* **6**, 163-166, doi:10.1016/j.aimed.2018.12.001 (2019).

21 Bidwell, A. J., Yazel, B., Davin, D., Fairchild, T. J. & Kanaley, J. A. Yoga training improves quality of life in women with asthma. *J Altern Complement Med* **18**, 749-755, doi:10.1089/acm.2011.0079 (2012).

22 Bruton, A. *et al.* Physiotherapy breathing retraining for asthma: a randomised controlled trial. *The Lancet Respiratory Medicine* **6**, 19-28, doi:10.1016/S2213-2600(17)30474-5 (2018).

23 Aparecido da Silva, R. *et al.* Constant-Load Exercise Versus High-Intensity Interval Training on Aerobic Fitness in Moderate-to-Severe Asthma: A Randomized Controlled Trial. *J Allergy Clin Immunol Pract* **10**, 2596-2604.e2597, doi:10.1016/j.jaip.2022.05.023 (2022).

24 Cooper, S. *et al.* Effect of two breathing exercises (Buteyko and pranayama) in asthma: A randomised controlled trial. *Thorax* **58**, 674-679, doi:10.1136/thorax.58.8.674 (2003).

25 Duruturk, N., Acar, M. & Doğrul, M. I. Effect of Inspiratory Muscle Training in the Management of Patients with Asthma A RANDOMIZED CONTROLLED TRIAL. *Journal of Cardiopulmonary Rehabilitation and Prevention* **38**, 198-203, doi:10.1097/HCR.0000000000000318 (2018).

26 Evaristo, K. B. *et al.* Effects of Aerobic Training Versus Breathing Exercises on Asthma Control: A Randomized Trial. *J Allergy Clin Immunol Pract* **8**, 2989-2996.e2984, doi:10.1016/j.jaip.2020.06.042 (2020).

27 Hiles, S. A., Urroz, P. D., Gibson, P. G., Bogdanovs, A. & McDonald, V. M. A feasibility randomised controlled trial of Novel Activity Management in severe ASthma-Tailored Exercise (NAMASTE): yoga and mindfulness. *BMC Pulmonary Medicine* **21**, doi:10.1186/s12890-021-01436-3 (2021).

28 Lage, S. M. *et al.* Efficacy of inspiratory muscle training on inspiratory muscle function, functional capacity, and quality of life in patients with asthma: A randomized controlled trial. *Clin Rehabil* **35**, 870-881, doi:10.1177/0269215520984047 (2021).

29 Mendes, F. A. R. *et al.* Effects of Aerobic Training on Psychosocial Morbidity and Symptoms in Patients With Asthma A Randomized Clinical Trial. *Chest* **138**, 331-337, doi:10.1378/chest.09-2389 (2010).

30 Prem, V., Sahoo, R. C. & Adhikari, P. Comparison of the effects of Buteyko and pranayama breathing techniques on quality of life in patients with asthma - a randomized controlled trial. *Clin Rehabil* **27**, 133-141, doi:10.1177/0269215512450521 (2013).

31 Alyse B, S. *et al.* Yoga intervention for adults with mild-to-moderate asthma: a pilot study. *Ann Allergy Asthma Immunol* **94**, doi:10.1016/s1081-1206(10)61131-3 (2005).

32 Thomas, M. *et al.* Breathing retraining for dysfunctional breathing in asthma: A randomised controlled trial. *Thorax* **58**, 110-115, doi:10.1136/thorax.58.2.110 (2012).

33 Toennesen, L. L. *et al.* Effects of Exercise and Diet in Nonobese Asthma Patients-A Randomized Controlled Trial. *Journal of Allergy and Clinical Immunology-in Practice* **6**, 803-811, doi:10.1016/j.jaip.2017.09.028 (2018).

34 Manocha, R., Marks, G. B., Kenchington, P., Peters, D. & Salome, C. M. Sahaja yoga in the management of moderate to severe asthma: a randomised controlled trial. *Thorax* **57**, 110-115, doi:10.1136/thorax.57.2.110 (2002).

35 Andreasson, K. H. *et al.* Breathing Exercises for Patients with Asthma in Specialist Care A Multicenter Randomized Clinical Trial. *Annals of the American Thoracic Society* **19**, 1498-1506, doi:10.1513/AnnalsATS.202111-1228OC (2022).

36 Zaryyab, H. Z., Shah, S. R., Saeed, S. & Anwar, N. in *Med. Forum.* 145.
37 RC, G. & MA, M. Effects of an aerobic physical training program on psychosocial characteristics, quality-of-life, symptoms and exhaled nitric oxide in individuals with moderate or severe persistent asthma. *Brazilian Journal of Physical Therapy* **12** (2008).
38 Refaat, A. & Gawish, M. Effect of physical training on health-related quality of life in patients with moderate and severe asthma. *Egyptian Journal of Chest Diseases and Tuberculosis* **64**, 761-766, doi:10.1016/j.ejcdt.2015.07.004 (2015).
39 Scott, H. *et al.* Dietary restriction and exercise improve airway inflammation and clinical outcomes in overweight and obese asthma: a randomized trial. *Clinical & Experimental Allergy* **43**, 36-49 (2013).
40 Holloway, E. A. & West, R. J. Integrated breathing and relaxation training (the Papworth method) for adults with asthma in primary care: a randomised controlled trial. *Thorax* **62**, 1039-1042, doi:10.1136/thx.2006.076430 (2007).
41 Coulson, E., Carpenter, L. M., Georgia, T. E. & Baptist, A. P. Breathing exercises in older adults with asthma: a blinded, randomized, placebo-controlled trial. *J Asthma* **59**, 1438-1444, doi:10.1080/02770903.2021.1936015 (2022).
42 Sodhi, C., Singh, S. & Dandona, P. K. A study of the effect of yoga training on pulmonary functions in patients with bronchial asthma. *Indian Journal of Physiology and Pharmacology* **53**, 169-174 (2009).
43 Sodhi, C., Singh, S. & Bery, A. Assessment of the quality of life in patients with bronchial asthma, before and after yoga: a randomised trial. *Iran J Allergy Asthma Immunol* **13**, 55-60 (2014).
44 Cowie, R. L., Conley, D. P., Underwood, M. F. & Reader, P. G. A randomised controlled trial of the Buteyko technique as an adjunct to conventional management of asthma. *Respiratory Medicine* **102**, 726-732, doi:10.1016/j.rmed.2007.12.012 (2008).
45 Shruti, A., Surya, K., Satyendra Kumar, M. & Ajay, V. Assessment of significance of Yoga on quality of life in asthma patients: A randomized controlled study. *Ayu* **38**, doi:10.4103/ayu.AYU_3_16 (2017).
46 Andreas, M., Sabine, G., Timm, V., Karin, T. & Hans J, B. A 12-month, moderate-intensity exercise training program improves fitness and quality of life in adults with asthma: a controlled trial. *BMC Pulm Med* **15**, doi:10.1186/s12890-015-0053-8 (2015).

47 Patricia D, F. *et al.* The Role of Exercise in a Weight-Loss Program on Clinical Control in Obese Adults with Asthma. A Randomized Controlled Trial. *Am J Respir Crit Care Med* **195**, doi:10.1164/rccm.201603-0446OC (2016).

Supplementary Information 3: Search Strategy

PARTICIPANTS	Asthma* [MESH] OR "Bronchial Spasm" [MESH] OR "Bronchoconstriction" [MESH]
INTERVENTION	1# (((((((((("Exercise"[Mesh] OR "Circuit-Based Exercise"[Mesh] OR "Cool-Down Exercise"[Mesh] OR "Exercise Therapy"[Mesh] OR "Resistance Training"[Mesh] OR "Muscle Stretching Exercises"[Mesh]) OR ("Endurance Training"[Mesh] OR "High-Intensity Interval Training"[Mesh])) OR "Running"[Mesh]) OR "Physical Conditioning, Human"[Mesh]) OR "breathing exercise"[Mesh]) OR "Yoga"[Mesh]) OR "Ventilation practice"[Mesh]) OR "Exercise Movement Techniques"[Mesh]) OR "Dance Therapy"[Mesh]) OR "Swimming"[Mesh]) OR "Bicycling"[Mesh]) OR "Walking"[Mesh]<br>2# (((((((strength train*[Title/Abstract]) OR (musc* training[Title/Abstract])) OR (endurance train*[Title/Abstract])) OR (aerobic train*[Title/Abstract])) OR (aerobic exercise*[Title/Abstract])) OR (kinesitherap*[Title/Abstract])) OR (continuous training[Title/Abstract])) OR (concurrent training[Title/Abstract])<br>1# OR 2#
OUTCOMES	"Quality of Life"[Mesh]
STUDY DESING	1# (((Randomized Controlled Trial[ptyp])) OR ((Controlled Clinical Trial[ptyp])) OR ((Clinical Trial[ptyp])) OR ("Clinical Trials as Topic"[Mesh]) OR ("Clinical Trials, Phase III as Topic"[Mesh]) OR ("Clinical Trials, Phase IV as Topic"[Mesh]) OR ("Controlled Clinical Trials as Topic"[Mesh]) OR ("Clinical Trial"[Publication Type]) OR ("Controlled Clinical Trial"[Publication Type]) OR ("Clinical Trial, Phase III"[Publication Type]) OR

	("Clinical Trial, Phase IV"[Publication Type]) OR ("Multicenter Study"[Publication Type]) OR ("Multicenter Studies as Topic"[Mesh]) OR ("Random Allocation"[Mesh]) OR ("Double-Blind Method"[Mesh]) OR ("Single-Blind Method"[Mesh]) OR ("Cross-Over Studies"[Mesh]) OR ("Placebos"[Mesh]) OR (controlled[Title/Abstract] AND (trial[Title/Abstract] OR trials[Title/Abstract] OR study[Title/Abstract] OR studies[Title/Abstract])) OR (blind[Title/Abstract] OR blinding[Title/Abstract] OR blinded[Title/Abstract] OR mask[Title/Abstract] OR masking[Title/Abstract] OR masked[Title/Abstract] OR placebo[Title/Abstract] OR placebos[Title/Abstract] OR rct[Title/Abstract] OR random[Title/Abstract] OR randomised[Title/Abstract] OR randomized[Title/Abstract] OR randomly[Title/Abstract] OR randomisation[Title/Abstract] OR randomization[Title/Abstract]) OR (factorial[Title/Abstract]) OR (divided[Title/Abstract] AND (group[Title/Abstract] OR groups[Title/Abstract])) OR (crossover[Title/Abstract]) OR ("cross over"[Title/Abstract]) OR (multicentre[Title/Abstract] OR multicentred[Title/Abstract] OR multicentric[Title/Abstract]) OR (versus[ti] OR vs[ti]) OR ("treatment arm"[Title/Abstract]) OR ("phase III"[Title/Abstract] OR "phase three"[Title/Abstract] OR "phase 3"[Title/Abstract]) OR ("latin square"[Title/Abstract]) NOT (("Animals"[Mesh] OR mouse[Title] OR mice[Title] OR pig[Title] OR pigs[Title] OR rat[Title] OR rats[Title] OR rabbit*[Title]) NOT (("Animals"[Mesh] OR mouse[Title] OR mice[Title] OR pig[Title] OR pigs[Title] OR rat[Title] OR rats[Title] OR rabbit*[Title] OR cadaver[Title] OR cadavers[Title]) AND "Humans"[Mesh])))) 2# ((“Non-Randomized Controlled Trials as Topic”[Mesh] OR (quasiexperiment* OR quasi experiment*).Title/Abstract)) 1# OR 2#
Note: Keyword groups will be linked by AND	

Search strategy for PubMed (MEDLINE)

PARTICIPANTS	3# 'asthma'/exp OR 'bronchospasm'/exp OR 'bronchoconstriction'/exp
INTERVENTION	1# 'exercise'/exp OR 'aerobic exercise'/exp OR 'aquatic exercise'/exp OR 'circuit training'/exp OR 'high intensity interval training'/exp OR 'interval training'/exp OR 'sprint interval training'/exp OR 'resistance training'/exp OR 'weight training'/exp OR 'power training'/exp OR 'exergaming'/exp OR 'low intensity exercise'/exp OR 'pilates'/exp OR 'breathing exercise'/exp OR 'tai chi'/exp OR 'yoga'/exp OR 'cycling'/exp OR 'walking'/exp OR 'swimming'/exp<br>2# 'strength training':ab,ti OR 'concurrent training':ab,ti OR 'musc training':ab,ti<br>1# OR 2#
OUTCOMES	4# 'quality of life'/exp
STUDY DESING	5# 'randomized controlled trial'/exp OR 'controlled clinical trial'/exp OR 'clinical trial'/exp OR 'clinical trial (topic)'/exp OR 'phase 3 clinical trial (topic)'/exp OR 'phase 4 clinical trial (topic)'/exp OR 'controlled clinical trial (topic)'/exp OR 'double blind procedure'/exp OR 'single blind procedure'/exp OR 'crossover procedure'/exp OR 'placebo'/exp
Note: Keyword groups will be linked by AND	

Search strategy for EMBASE

PARTICIPANTS	((TS=(Asthma*)) OR TS=(Bronchial Spasm)) OR TS=(Bronchoconstriction)
INTERVENTION	((((((((((((((((TS=(Exercise*)) OR TS=(Circuit-Based Exercise)) OR TS=(aerobic exercise)) OR TS=(aquatic exercise)) OR TS=(high intensity interval training)) OR TS=(interval training)) OR TS=(resistance training)) OR TS=(swimming)) OR TS=(walking)) OR TS=(cycling)) OR TS=(yoga)) OR TS=(breathing exercise)) OR TS=(stretching exercise)) OR TS=(concurrent training)) OR TS=(musc* training)) OR TS=(train*)) OR TS=(strength training)) OR TS=(Steady State Training)

OUTCOMES	TS=(quality of life)
STUDY DESING	((((((TS=(randomised)) OR TS=(randomized)) OR TS=(randomisation)) OR TS=(randomisation)) OR TS=(placebo)) OR (TS=(random)) AND (TS=(allocat)) OR TS=(assign)) OR (TS=(blind)) AND (((TS=(single)) OR TS=(double)) OR TS=(treble)) OR TS=(triple)
Note: Keyword groups will be linked by AND	

Search strategy for Web of science

PARTICIPANTS	#1 MeSH descriptor: [Asthma] explode all trees<br>#2 MeSH descriptor: [Bronchial Spasm] explode all trees<br>#3 MeSH descriptor: [Bronchoconstriction] explode all trees<br>#4 (#1 OR #2 OR #3)
INTERVENTION	#5 MeSH descriptor: [Exercise] explode all trees<br>#6 MeSH descriptor: [Muscle Stretching Exercises] explode all trees<br>#7 MeSH descriptor: [Endurance Training] explode all trees<br>#8 MeSH descriptor: [Circuit-Based Exercise] explode all trees<br>#9 MeSH descriptor: [High-Intensity Interval Training] explode all trees<br>#10 MeSH descriptor: [Physical Conditioning, Human] explode all trees<br>#11 MeSH descriptor: [Preoperative Exercise] explode all trees<br>#12 MeSH descriptor: [Running] explode all trees

	#13 MeSH descriptor: [Swimming] explode all trees<br>#14 MeSH descriptor: [Walking] explode all trees<br>#15 MeSH descriptor: [Exercise Therapy] explode all trees<br>#16 MeSH descriptor: [Physical Conditioning, Human] explode all trees<br>#17 MeSH descriptor: [breathing exercise] explode all trees<br>#18 MeSH descriptor: [Yoga] explode all trees<br>#19 MeSH descriptor: [Bicycling] explode all trees<br>#20 (#5 OR #6 OR #7 OR #8 OR #9 OR #10 OR 11 0R 12 0R #13 0R #14 0R #15 0R #16 0R #17 0R #18 0R #19)<br>#21 (strength train).i OR (musc training)ti,ab,kw OR (endurance rain*):ti,ab kw OR (aerobic train*).ti.ab,kw OR (concurrent raining)t.ab.kw<br>#22 (#20 OR #21)
OUTCOMES	#23 MeSH descriptor: [Respiratory Function Tests] explode all trees<br>#24 MeSH descriptor: [Quality of Life] explode all trees<br>#25 MeSH descriptor: [Exercise Test] explode all trees<br>#26 MeSH descriptor: [Physical Fitness] explode all trees<br>#27 (#23 OR #24 OR #25 OR #26)
STUDY DESING	#28 MeSH descriptor: [Randomized Controlled Trial] explode all trees<br>#29 MeSH descriptor: [Controlled Clinical Trial] explode all trees

	#30 MeSH descriptor: [Controlled Clinical Trials as Topic] explode all trees<br><br>#31 MeSH descriptor: [Random Allocation] explode all trees<br><br>#32 MeSH descriptor: [Double-Blind Method] explode all trees<br><br>#33 MeSH descriptor:[Single-Blind Method] explode all trees<br><br>#34 MeSH descriptor: [Cross-Over Studies] explode all trees<br><br>#35 MeSH descriptor: [Placebos] explode all trees<br><br>#36 (#28 OR #29 OR #30 OR #31 OR #32 OR #33 OR #34 0R #35)
Note: Keyword groups will be linked by AND	

Search strategy for Cochrane Central Register of Controlled Trials

Supplementary Information 4: classification and definition of exercise types and non-exercise group

4.1 classification of exercise types and non-exercise group

The exercise program is categorized into two types (single-mode exercise and comprehensive-mode exercise) based on the including elements (Fig. 5.1). Single-mode exercise includes inspiratory muscle training, various breathing exercises (Diaphragmatic and nasal, pranayama, buteko and papworth breathing exercise), and Whole-body exercise (moderate-intensity aerobic exercise and high-intensity interval training); comprehensive-mode exercise includes yoga (composed of asanas, pranayama, and meditation), aerobic combined with breathing exercise, aerobic combined with resistance exercise.

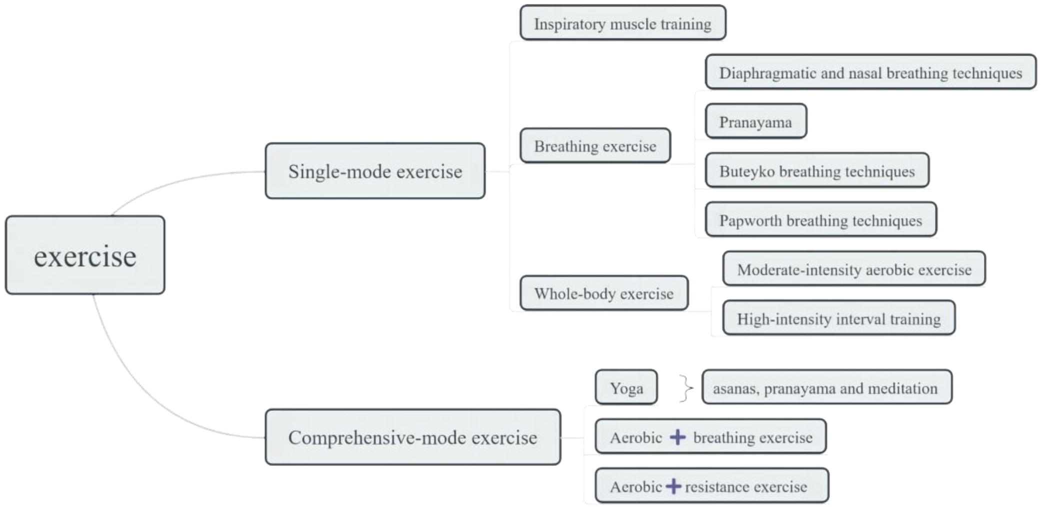

Figure 5.1 classification of exercise types

4.2 definition of exercise types and non-exercise group

abbreviation	Full name	Definitions
aerobic	Moderate-intensity aerobic exercise	Aerobic exercise is performed by activities for extended periods of time.[4] e.g., walking, bicycle, treadmill training etc.
HIIT	High-intensity interval training	High-Intensity Interval Training (HIIT) is a exercise training method characterized by alternating short periods of high-intensity exercise with rest or low-intensity exercise. HIIT training includes brief high-intensity exercises (such as sprinting or fast cycling) followed by relatively short periods of rest or low-intensity exercise (such as walking or slow cycling). This cycle of alternating intensity levels continues for a set period of time, with training sessions typically being short but highly effective [5].
CON	Control group	Non-exercise intervention, usual care, or asthma education
breathing	Diaphragmatic and nasal breathing exercise	Diaphragmatic breathing (DB) is slow and deep breathing that affects the brain and the cardiovascular, respiratory, and gastrointestinal systems through the modulation of

abbreviation	Full name	Definitions
		autonomic nervous functions [6].
pranayama	Pranayama breathing exercise	Pranayama is a breathing control technique in yoga. The main function of pranayama is to promote smooth breathing and unity of body and mind for practitioners [7].
Buteyko	Buteyko breathing exercise	The late Professor Konstantin Buteyko was a Russian physiologist who gave his name to a novel treatment approach that is currently being applied to patients with asthma in a number of countries. The approach varies in some details in different countries and with different practitioners, but essentially consists of a package of breathing therapy, relaxation techniques and exercises combined with advice and education about medication use, nutrition and general health [8].
papworth	Papworth breathing exercise	An integrated breathing and relaxation technique known as the Papworth method has been implemented by physiotherapists since the 1960s for patients with asthma and dysfunctional breathing [9].
aerobic_ breathing	Aerobic exercise combined with Breathing training	Aerobic exercise combined with Breathing training
aerobic_ resistance	Aerobic exercise combined with resistance training	Aerobic exercise combined with resistance training. resistance training designed to improve the strength, power, endurance and size of skeletal muscles [10].
IMT	Inspiratory muscle training	Inspiratory Muscle Training (IMT) is a training method that can improve inspiratory muscle strength in ICU patients. IMT is conducted using a handheld device that adjusts inspiratory resistance during the inspiratory phase to train the inspiratory muscles of patients [11].
Yoga	-	Mainly a series of methods for self-cultivation, including body-adjusting asanas (refer to yoga asana collection), breathing-adjusting breathing methods, and mind-adjusting meditation, etc., to achieve the unity of body and mind [12].

Supplementary Information 5: Results from network meta-analyses

The following shows the network plots and the league table of the secondary outcomes (QOL subdimension score).

5.1 symptoms

Fig 1A displays a network diagram of the qualified asthma QOL symptoms score comparisons. all exercise methods were compared with the control group at least once. Compared with the control group, all Comprehensive-mode exercise signifcantly improved the asthma QOL total score of participants, and the SMDs (95% Credible Interval (CrI)) ranged between 2.09 (1.62 to 2.56) for Yoga to 1.40 (0.24 to 2.55) for aerobic_resistance (Fig. 2A and Table 1A), and Yoga ranks frst (P-score = 0.89).

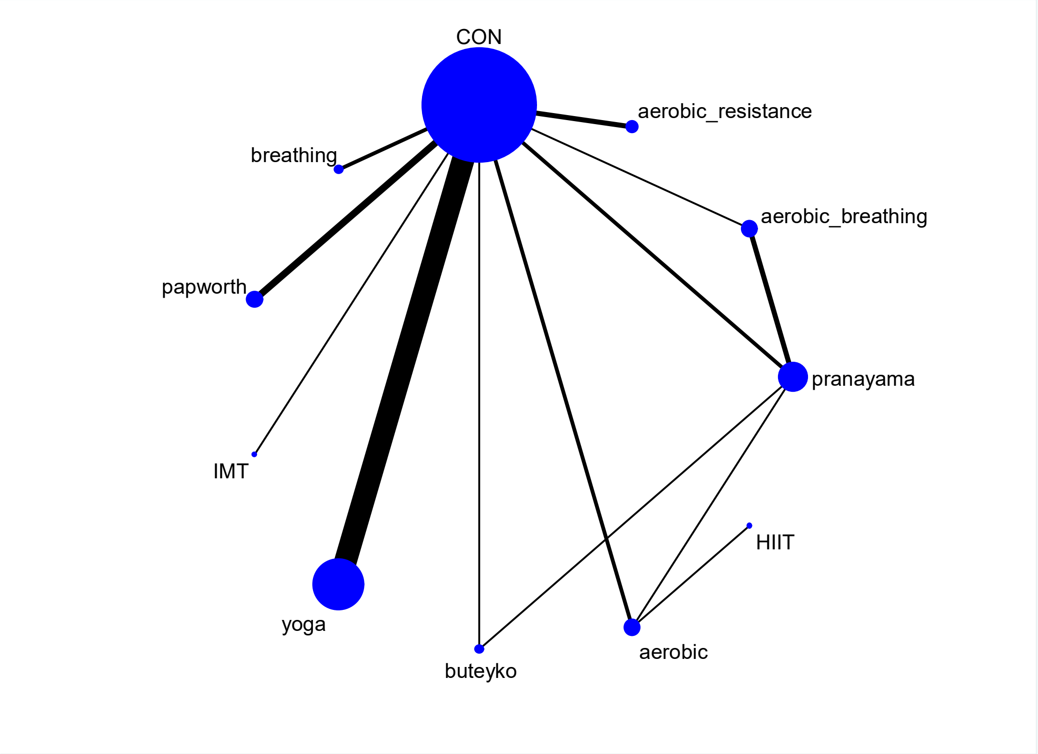

Figure 1A. Network plot of asthma QOL symptoms score. The size of the nodes corresponds to the number of participants randomized to each hypoxic training. Exercise type with direct comparisons are linked with a line; its thickness corresponds to the number of trials evaluating the comparison. CON “control group”; breathing “ diaphragm breathing exercsie”; papworth “Papworth breathing exercise”; IMT “inspiration muscle training”; buteyko “Buteyko breathing exercise”; aerobic moderate intensity aerobic exercise”; HIIT “High-intensity interval training”; pranayama “Pranayama breathing exercise”; aerobic_breathing “aerobic exercise combined with breathing exercise”; aerobic_resistance “aerobic exercise combined with resistance exercise”.

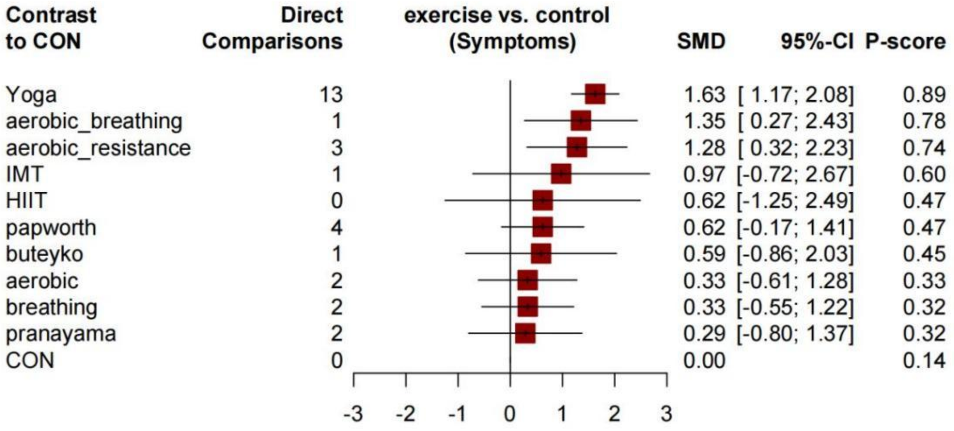

Fig. 2A Forest plot change in effect of asthma QOL symptoms score. Various LLTH modes are ranked according to the surface under the curved cumulative ranking probabilities. Treatments crossing the y-axis are not signifcantly diferent from CON. The n value represents the number of studies that were directly compared to the control group. SMD “standardized Mean Diference”; CrI “Credible Interval”; CON “control group”; breathing “ diaphragm breathing exercsie”; papworth “Papworth breathing

exercise”; IMT “inspiration muscle training”; buteyko “Buteyko breathing exercise”; aerobic moderate intensity aerobic exercise”; HIIT “High-intensity interval training”; pranayama “Pranayama breathing exercise”; aerobic_breathing “aerobic exercise combined with breathing exercise”; aerobic_resistance “aerobic exercise combined with resistance exercise”.

Yoga	.	.	.	.	.	.	.	.	.	**2.09 (1.62; 2.56)**
0.56 (-0.53; 1.66)	aerobic_breathing	.	.	.	.	.	.	.	.	**1.53 (0.54; 2.52)**
0.69 (-0.55; 1.94)	0.13 (-1.39; 1.65)	aerobic_resistance	.	.	.	.	.	.	**1.76 (0.58; 2.94)**	0.31 (-1.42; 2.04)
1.44 (-0.33; 3.21)	0.88 (-1.09; 2.85)	0.75 (-1.31; 2.81)	IMT	.	.	.	.	.	.	0.65 (-1.06; 2.35)
1.59 (-0.36; 3.55)	1.03 (-1.11; 3.17)	0.90 (-1.24; 3.04)	0.15 (-2.40; 2.70)	HIIT	.	.	0.31 (-1.32; 1.95)	.	.	.
**1.67 (0.13; 3.21)**	1.11 (-0.66; 2.88)	0.98 (-0.73; 2.69)	0.23 (-2.02; 2.48)	0.08 (-2.27; 2.42)	papworth	.	.	.	0.30 (-1.32; 1.91)	0.40 (-1.21; 2.01)
**1.71 (0.51; 2.91)**	1.15 (-0.33; 2.63)	1.02 (-0.58; 2.62)	0.27 (-1.76; 2.30)	0.12 (-2.08; 2.32)	0.04 (-1.79; 1.88)	buteyko	.	.	.	0.38 (-0.73; 1.48)
**1.91 (0.84; 2.97)**	1.34 (-0.03; 2.72)	1.21 (-0.16; 2.59)	0.47 (-1.49; 2.42)	0.31 (-1.32; 1.95)	0.24 (-1.44; 1.91)	0.19 (-1.27; 1.66)	aerobic	.	0.19 (-1.45; 1.83)	0.12 (-0.99; 1.22)
**1.97 (0.75; 3.18)**	1.40 (-0.09; 2.89)	1.27 (-0.33; 2.88)	0.53 (-1.51; 2.56)	0.37 (-1.83; 2.57)	0.29 (-1.55; 2.14)	0.25 (-1.31; 1.82)	0.06 (-1.41; 1.53)	breathing	.	0.12 (-0.99; 1.24)
**1.95 (0.93; 2.97)**	**1.39 (0.05; 2.73)**	**1.26 (0.24; 2.27)**	0.51 (-1.42; 2.44)	0.36 (-1.62; 2.33)	0.28 (-1.19; 1.75)	0.24 (-1.19; 1.66)	0.04 (-1.06; 1.15)	-0.02 (-1.45; 1.42)	pranayama	0.70 (-0.46; 1.86)
**2.09 (1.62; 2.56)**	**1.53 (0.54; 2.52)**	**1.40 (0.24; 2.55)**	0.65 (-1.06; 2.35)	0.50 (-1.40; 2.40)	0.42 (-1.05; 1.89)	0.38 (-0.73; 1.48)	0.18 (-0.78; 1.14)	0.12 (-0.99; 1.24)	0.14 (-0.76; 1.04)	CON

Table 1A. League table for changes in asthma QOL symptoms score associated with various exercise modes.

5.2 activity limitations

Fig 1B displays a network diagram of the qualified asthma QOL activity limitations score comparisons. all exercise methods were compared with the control group at least once. Compared with the control group, all Comprehensive-mode exercise signifcantly improved the asthma QOL total score of participants, and the SMDs (95% Credible Interval (CrI)) ranged between 2.24 (1.65 to 2.83) for Yoga to 1.38 (0.16 to 2.61) for aerobic_resistance (Fig. 2B and Table 1B), and Yoga ranks frst (P-score = 0.92).

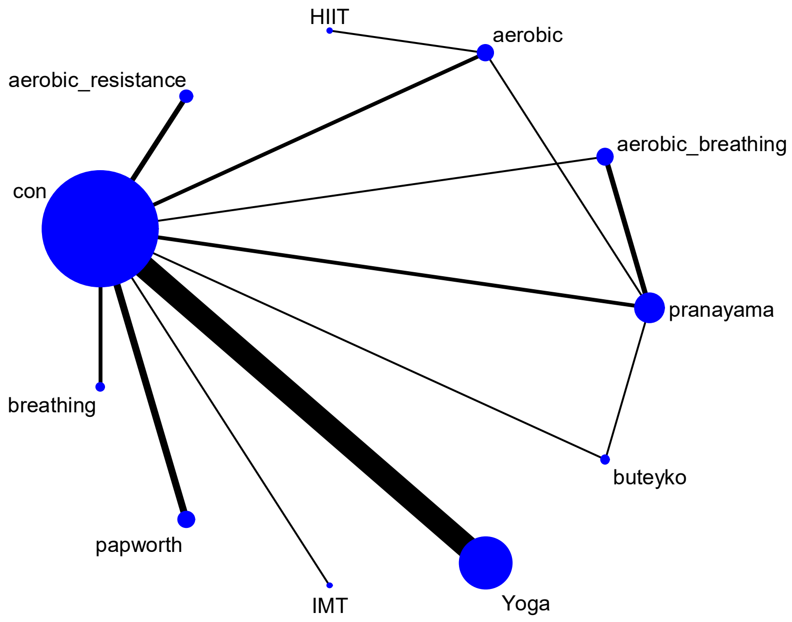

Figure 1B. Network plot of activity limitations QOL symptoms score. The size of the nodes corresponds to the number of participants randomized to each hypoxic training. Exercise type with direct comparisons are linked with a line; its thickness corresponds to the number of trials evaluating the comparison. CON "control group"; breathing " diaphragm breathing exercsie"; papworth "Papworth breathing exercise"; IMT "inspiration muscle training"; buteyko "Buteyko breathing exercise"; aerobic moderate

intensity aerobic exercise”; HIIT “High-intensity interval training”; pranayama “Pranayama breathing exercise”; aerobic_breathing “aerobic exercise combined with breathing exercise”; aerobic_resistance “aerobic exercise combined with resistance exercise”.

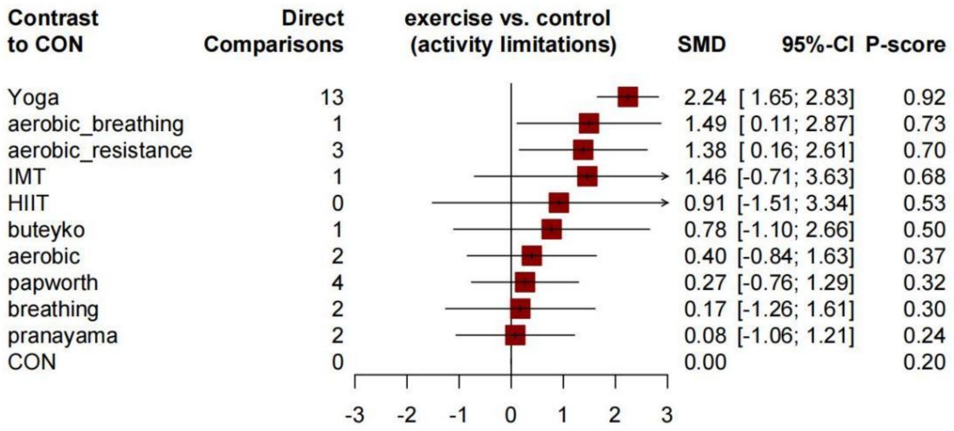

Fig. 2B Forest plot change in effect of activity limitations QOL symptoms score. Various LLTH modes are ranked according to the surface under the curved cumulative ranking probabilities. Treatments crossing the y-axis are not signifcantly diferent from CON. The n value represents the number of studies that were directly compared to the control group. SMD “standardized Mean Diference”; CrI “Credible Interval”; CON “control group”; breathing “ diaphragm breathing exercsie”; papworth “Papworth breathing exercise”; IMT “inspiration muscle training”; buteyko “Buteyko breathing exercise”; aerobic moderate intensity aerobic exercise”; HIIT “High-intensity interval training”; pranayama “Pranayama breathing exercise”; aerobic_breathing “aerobic exercise combined with breathing exercise”; aerobic_resistance “aerobic exercise combined with resistance exercise”.

Yoga	.	.	.	.	.	.	.	.	.	**2.24 (1.65; 2.83)**
0.75 (-0.75; 2.25)	aerobic_breathing	.	.	.	.	.	.	.	**1.66 (0.41; 2.91)**	0.75 (-1.43; 2.92)
0.86 (-0.50; 2.22)	0.11 (-1.74; 1.96)	aerobic_resistance	.	.	.	.	.	.	.	**1.38 (0.16; 2.61)**
0.78 (-1.47; 3.02)	0.03 (-2.54; 2.60)	-0.08 (-2.57; 2.41)	IMT	.	.	.	.	.	.	1.46 (-0.71; 3.63)
1.32 (-1.17; 3.82)	0.58 (-2.10; 3.26)	0.47 (-2.25; 3.19)	0.55 (-2.71; 3.80)	HIIT	.	0.52 (-1.57; 2.61)	.	.	.	.
1.46 (-0.51; 3.43)	0.72 (-1.40; 2.83)	0.61 (-1.64; 2.85)	0.69 (-2.18; 3.55)	0.14 (-2.86; 3.14)	Buteyko	.	.	.	0.42 (-1.65; 2.49)	1.05 (-1.02; 3.12)
**1.84 (0.47; 3.21)**	1.10 (-0.58; 2.78)	0.99 (-0.76; 2.73)	1.07 (-1.43; 3.56)	0.52 (-1.57; 2.61)	0.38 (-1.78; 2.54)	aerobic	.	.	0.91 (-1.18; 3.01)	0.12 (-1.32; 1.55)
**1.97 (0.79; 3.16)**	1.23 (-0.49; 2.95)	1.12 (-0.48; 2.72)	1.20 (-1.20; 3.59)	0.65 (-1.99; 3.29)	0.51 (-1.63; 2.65)	0.13 (-1.48; 1.74)	papworth	.	.	0.27 (-0.76; 1.29)
**2.06 (0.52; 3.61)**	1.32 (-0.67; 3.31)	1.21 (-0.68; 3.09)	1.29 (-1.31; 3.89)	0.74 (-2.08; 3.56)	0.60 (-1.76; 2.97)	0.22 (-1.67; 2.11)	0.09 (-1.67; 1.85)	breathing	.	0.17 (-1.26; 1.61)
**2.16 (0.88; 3.44)**	**1.41 (0.29; 2.53)**	1.30 (-0.37; 2.98)	1.38 (-1.06; 3.83)	0.84 (-1.68; 3.36)	0.70 (-1.18; 2.58)	0.32 (-1.10; 1.73)	0.19 (-1.34; 1.72)	0.10 (-1.73; 1.93)	pranayama	0.71 (-0.76; 2.19)
**2.24 (1.65; 2.83)**	**1.49 (0.11; 2.87)**	**1.38 (0.16; 2.61)**	1.46 (-0.71; 3.63)	0.91 (-1.51; 3.34)	0.78 (-1.10; 2.66)	0.40 (-0.84; 1.63)	0.27 (-0.76; 1.29)	0.17 (-1.26; 1.61)	0.08 (-1.06; 1.21)	CON

Table 1B. League table for changes in activity limitations QOL symptoms score associated with various exercise modes.

5.3 Emotional Functions

Fig 1C displays a network diagram of the qualified asthma QOL Emotional Functions score comparisons. all exercise methods were compared with the control group at least once. Compared with the control group, all Comprehensive-mode exercise signifcantly improved the asthma QOL total score of participants, and the SMDs (95% Credible Interval (CrI)) ranged between 2.09 (1.62 to 2.56) for Yoga to 1.40 (0.24 to 2.55) for aerobic_breathing (Fig. 2C and Table 1C), and Yoga ranks frst (P-score = 0.96).

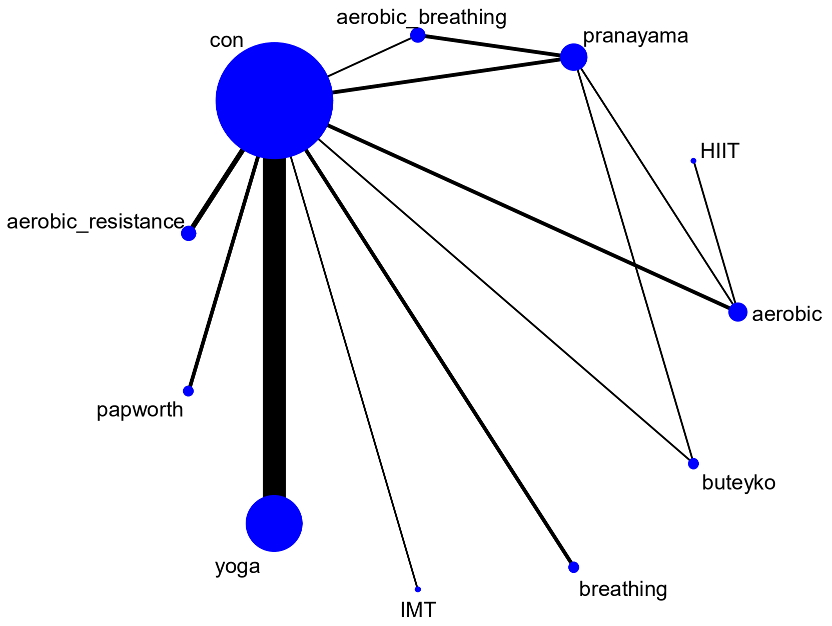

Figure 1C. Network plot of Emotional Functions QOL symptoms score. The size of the nodes corresponds to the number of participants randomized to each hypoxic training. Exercise type with direct comparisons are linked with a line; its thickness corresponds to the number of trials evaluating the comparison. CON "control group"; breathing " diaphragm breathing exercsie"; papworth "Papworth breathing exercise"; IMT "inspiration muscle training"; buteyko "Buteyko breathing exercise"; aerobic moderate

intensity aerobic exercise”; HIIT “High-intensity interval training”; pranayama “Pranayama breathing exercise”; aerobic_breathing “aerobic exercise combined with breathing exercise”; aerobic_resistance “aerobic exercise combined with resistance exercise”.

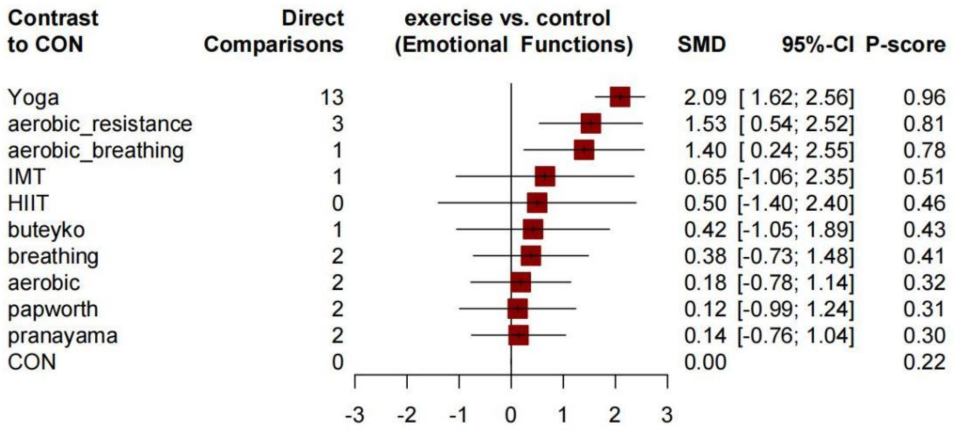

Fig. 2C Forest plot change in effect of Emotional Functions QOL symptoms score. Various LLTH modes are ranked according to the surface under the curved cumulative ranking probabilities. Treatments crossing the y-axis are not signifcantly diferent from CON. The n value represents the number of studies that were directly compared to the control group. SMD “standardized Mean Diference”; CrI “Credible Interval”; CON “control group”; breathing “ diaphragm breathing exercsie”; papworth “Papworth breathing exercise”; IMT “inspiration muscle training”; buteyko “Buteyko breathing exercise”; aerobic moderate intensity aerobic exercise”; HIIT “High-intensity interval training”; pranayama “Pranayama breathing exercise”; aerobic_breathing “aerobic exercise combined with breathing exercise”; aerobic_resistance “aerobic exercise combined with resistance exercise”.

Yoga	.	.	.	.	.	.	.	.	.	**2.09 (1.62; 2.56)**
0.56 (-0.53; 1.66)	aerobic_resistance	.	.	.	.	.	.	.	.	**1.53 (0.54; 2.52)**
0.69 (-0.55; 1.94)	0.13 (-1.39; 1.65)	aerobic_breathing	.	.	.	.	.	.	**1.76 (0.58; 2.94)**	0.31 (-1.42; 2.04)
1.44 (-0.33; 3.21)	0.88 (-1.09; 2.85)	0.75 (-1.31; 2.81)	IMT	.	.	.	.	.	.	0.65 (-1.06; 2.35)
1.59 (-0.36; 3.55)	1.03 (-1.11; 3.17)	0.90 (-1.24; 3.04)	0.15 (-2.40; 2.70)	HIIT	.	.	0.31 (-1.32; 1.95)	.	.	.
**1.67 (0.13; 3.21)**	1.11 (-0.66; 2.88)	0.98 (-0.73; 2.69)	0.23 (-2.02; 2.48)	0.08 (-2.27; 2.42)	buteyko	.	.	.	0.30 (-1.32; 1.91)	0.40 (-1.21; 2.01)
**1.71 (0.51; 2.91)**	1.15 (-0.33; 2.63)	1.02 (-0.58; 2.62)	0.27 (-1.76; 2.30)	0.12 (-2.08; 2.32)	0.04 (-1.79; 1.88)	breathing	.	.	.	0.38 (-0.73; 1.48)
**1.91 (0.84; 2.97)**	1.34 (-0.03; 2.72)	1.21 (-0.16; 2.59)	0.47 (-1.49; 2.42)	0.31 (-1.32; 1.95)	0.24 (-1.44; 1.91)	0.19 (-1.27; 1.66)	aerobic	.	0.19 (-1.45; 1.83)	0.12 (-0.99; 1.22)
**1.97 (0.75; 3.18)**	1.40 (-0.09; 2.89)	1.27 (-0.33; 2.88)	0.53 (-1.51; 2.56)	0.37 (-1.83; 2.57)	0.29 (-1.55; 2.14)	0.25 (-1.31; 1.82)	0.06 (-1.41; 1.53)	papworth	.	0.12 (-0.99; 1.24)
**1.95 (0.93; 2.97)**	**1.39 (0.05; 2.73)**	**1.26 (0.24; 2.27)**	0.51 (-1.42; 2.44)	0.36 (-1.62; 2.33)	0.28 (-1.19; 1.75)	0.24 (-1.19; 1.66)	0.04 (-1.06; 1.15)	-0.02 (-1.45; 1.42)	pranayama	0.70 (-0.46; 1.86)
**2.09 (1.62; 2.56)**	**1.53 (0.54; 2.52)**	**1.40 (0.24; 2.55)**	0.65 (-1.06; 2.35)	0.50 (-1.40; 2.40)	0.42 (-1.05; 1.89)	0.38 (-0.73; 1.48)	0.18 (-0.78; 1.14)	0.12 (-0.99; 1.24)	0.14 (-0.76; 1.04)	CON

Table 1C. League table for changes in Emotional Functions QOL symptoms score associated with various exercise modes.

5.4 environmental stimulants

Fig 1D displays a network diagram of the qualified asthma QOL environmental stimulants score comparisons. all exercise methods were compared with the control group at least once. Compared with the control group, all Comprehensive-mode exercise signifcantly improved the asthma QOL total score of participants, and the SMDs (95% Credible Interval (CrI)) ranged between 1.39 (0.17 to 2.61) for aerobic_breathing to 1.07 (0.64 to 1.50) for yoga (Fig. 2D and Table 1D), and Yoga ranks frst (P-score = 0.96).

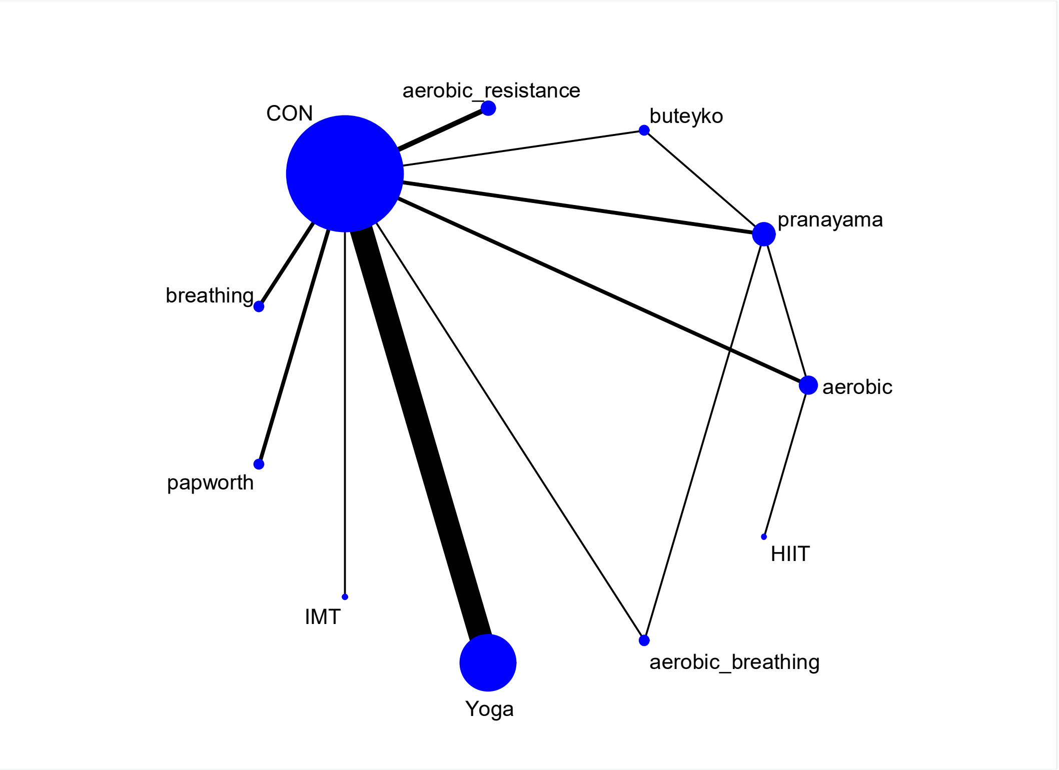

Figure 1D. Network plot of environmental stimulants QOL symptoms score. The size of the nodes corresponds to the number of participants randomized to each hypoxic training. Exercise type with direct comparisons are linked with a line; its thickness corresponds to the number of trials evaluating the comparison. CON "control group";

breathing " diaphragm breathing exercsie"; papworth "Papworth breathing exercise"; IMT "inspiration muscle training"; buteyko "Buteyko breathing exercise"; aerobic moderate intensity aerobic exercise"; HIIT "High-intensity interval training"; pranayama "Pranayama breathing exercise"; aerobic_breathing "aerobic exercise combined with breathing exercise"; aerobic_resistance "aerobic exercise combined with resistance exercise".

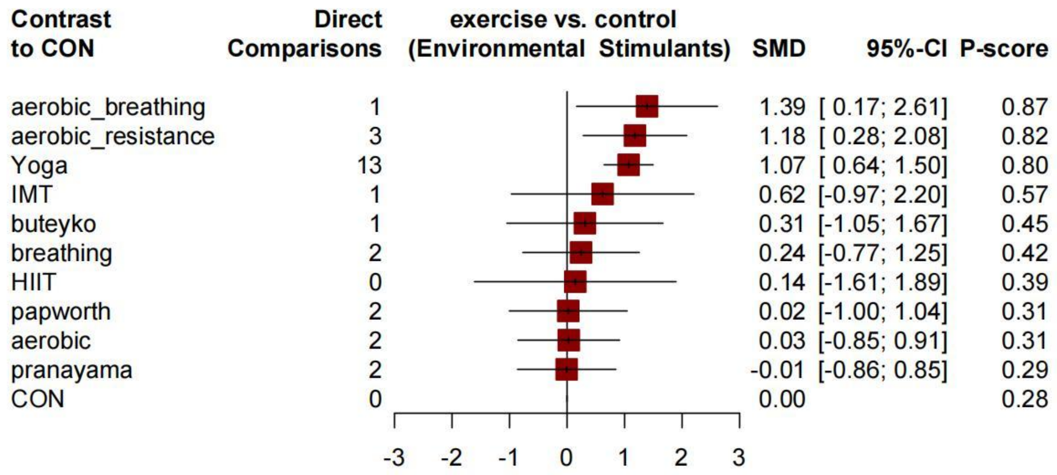

Fig. 2D Forest plot change in effect of environmental stimulants QOL symptoms score. Various LLTH modes are ranked according to the surface under the curved cumulative ranking probabilities. Treatments crossing the y-axis are not signifcantly diferent from CON. The n value represents the number of studies that were directly compared to the control group. SMD "standardized Mean Diference"; CrI "Credible Interval"; CON "control group"; breathing " diaphragm breathing exercsie"; papworth

“Papworth breathing exercise”; IMT “inspiration muscle training”; buteyko “Buteyko breathing exercise”; aerobic moderate intensity aerobic exercise”; HIIT “High-intensity interval training”; pranayama “Pranayama breathing exercise”; aerobic_breathing “aerobic exercise combined with breathing exercise”; aerobic_resistance “aerobic exercise combined with resistance exercise”.

Supplementary Information 6: Statistical methods in details

Network meta-analysis

We will use R software gemtc and rjags packages to perform Bayesian network meta-analysis. Using arm-level data and import into the R software in CSV format. The effect size measure for continuous outcomes chooses the standardized mean difference (SMD) of the change score (end-point minus baseline score) because the studies use different rating scales or units of clinician disability, motor outcomes, non-motor outcomes, muscle strength, and concern of falling. The normal likelihood for continuous outcomes. The study effect sizes were then synthesized using a random-effects network meta-analysis model. In addition, we will present the summary SMD, 95% credible intervals (CrIs) for all pairwise comparisons in the league table, and we show the results of comparing the outcomes of each exercise intervention group and the control group in the form of a forest plot. To rank the various treatments for each outcome, we will use the surface under the cumulative ranking curve (SUCRA). In the process of extracting data, if the original study reported a standard error in the experimental and control groups, the standard deviation was calculated by the formula: standard deviation (SD) = standard error (SE) × √n. If both are missing, we will estimate SD based on the confidence interval, t-value, quartile, range, or p-values as described in section 7.7.3 of the Cochrane Handbook for Systematic Reviews. When only figures were presented, data were extracted using GetData (http://getdata-graph-digitizer.com) to measure the length (in pixels) of the axes to calibrate and then the length in pixels from the relevant axis to the data points of interest. If the data needed for the study cannot be extracted from the above methods, we will ask the authors about the data at least 4 times within 6 weeks.

Continuous variable-random effects model:

For any physical activity intervention x in any randomized controlled experiment i, the sample size is $n_{i,\,x}$. The effect of treatment is $y_{i,\,x}$ (change from baseline), and standard error is $se_{i,\,x}$. Then the normal
likelihood is employed to $y_{i,\,x} \sim N\ (\theta_{i,\,y},\ se_{i,\,x})$ in each arm. In addition, for any randomized controlled experiment, there should be a basic physical activity type b(i), and its effect is represented by $y_{i,\,b(i)}$

$$y_{i,\,b(i)} = u_i$$

In the random effects model, for any interventional physical activity k that is not a basic physical activity type, its exercise effect is:

$$\theta_{i,\,k} = u_i + \delta_{i,\,b(i),\,k} \qquad \text{for } k \geq 2$$

Where $\delta_{i,\,b(i),\,k}$ is the difference between the effect of physical activity k and basic physical activity b, and conforms to the following normal distribution $\delta_{i,\,x,\,y} \sim N(d_{x,y},\ \boldsymbol{\sigma}^2_{x,\,y})$, where $d_{x,y}$ is the relative effect of physical activity intervention y and x, $\boldsymbol{\sigma}^2_{x,\,y}$ is the variance of the relative effect of physical activity intervention y and x. In addition, this study presented the final treatment effect with standardised mean difference (SMD), so the above formula is modified:

$$y_{i,\,b(i)} = u_i/S_i$$

$$\theta_{i,\,k} = (u_i + \delta_{i,\,b(i),\,y})/\ S_i \qquad \text{for } k \geq 2$$

where S_i is the pooled standard deviation in the study arms

```
model {
 # Likelihood for arm-based data
 ## OMITTED
 # Likelihood for contrast-based data (univariate for 2-arm trials)
 for(i in studies.r2) {
        for (k in 2:na[i]) {
               mest[i, k] <- delta[i, k]
        }
        m[i, 2] ~ dnorm(mest[i, 2], prec[i, 2])
        prec[i, 2] <- 1 / (e[i, 2] * e[i, 2])

        dev[i, 1] <- pow(m[i, 2] - mest[i, 2], 2) * prec[i, 2]
 }
 # Likelihood for contrast-based data (multivariate for multi-arm trials)
 for(i in studies.rm) {
        for (k in 2:na[i]) {
               mest[i, k] <- delta[i, k]
        }
        for (k in 1:(na[i]-1)) {
               for (j in 1:(na[i]-1)) {
                      Sigma[i,j,k] <- ifelse(equals(j, k), pow(e[i,k+1], 2), pow(e[i,1], 2))
               }
        }
        Omega[i,1:(na[i]-1),1:(na[i]-1)] <- inverse(Sigma[i,1:(na[i]-1),1:(na[i]-1)])
        m[i,2:na[i]] ~ dmnorm(mest[i,2:na[i]], Omega[i,1:(na[i]-1),1:(na[i]-1)])

        mdiff[i, 2:na[i]] <- m[i, 2:na[i]] - mest[i, 2:na[i]]
        dev[i, 1] <- t(mdiff[i, 2:na[i]]) %*% Omega[i, 1:(na[i]-1),1:(na[i]-1)] %*% mdiff[i,
2:na[i]]
 }

 # Random effects model
 for (i in studies) {
        # Study-level relative effects
        w[i, 1] <- 0
        delta[i, 1] <- 0
        for (k in 2:na[i]) { # parameterize multi-arm trials using a trick to avoid dmnorm
               delta[i, k] ~ dnorm(md[i, k], taud[i, k])
               md[i, k] <- d[t[i, 1], t[i, k]] + sw[i, k]
               taud[i, k] <- tau.d * 2 * (k - 1) / k
               w[i, k] <- delta[i, k] - (d[t[i, 1], t[i, k]])
               sw[i, k] <- sum(w[i, 1:(k-1)]) / (k - 1)
        }
```

```
}

# Random effects variance prior
sd.d ~ dunif(0, om.scale)
tau.d <- pow(sd.d, -2)

# Relative effect matrix
d[1, 1] <- 0
d[1, 2] <- -d.CON.aerobic + d.CON.aerobic_breathing
d[1, 3] <- -d.CON.aerobic + d.CON.aerobic_resistance
d[1, 4] <- -d.CON.aerobic + d.CON.breathing
d[1, 5] <- -d.CON.aerobic + d.CON.Buteyk
d[1, 6] <- -d.CON.aerobic
d[1, 7] <- -d.CON.aerobic + d.CON.HIIT
d[1, 8] <- -d.CON.aerobic + d.CON.IMT
d[1, 9] <- -d.CON.aerobic + d.CON.Papworth
d[1, 10] <- -d.CON.aerobic + d.CON.pranayama
d[1, 11] <- -d.CON.aerobic + d.CON.Yoga
for (i in 2:nt) {
        for (j in 1:nt) {
                d[i, j] <- d[1, j] - d[1, i]
        }
}

prior.prec <- pow(re.prior.sd, -2)

# Study baseline priors
## OMITTED

# Effect parameter priors
d.CON.aerobic ~ dnorm(0, prior.prec)
d.CON.aerobic_breathing ~ dnorm(0, prior.prec)
d.CON.aerobic_resistance ~ dnorm(0, prior.prec)
d.CON.breathing ~ dnorm(0, prior.prec)
d.CON.Buteyk ~ dnorm(0, prior.prec)
d.CON.HIIT ~ dnorm(0, prior.prec)
d.CON.IMT ~ dnorm(0, prior.prec)
d.CON.Papworth ~ dnorm(0, prior.prec)
d.CON.pranayama ~ dnorm(0, prior.prec)
d.CON.Yoga ~ dnorm(0, prior.prec)
```

Assessment of the transitivity assumption

Different clinical trials need to ensure that their baseline levels are consistent. If the baseline levels are inconsistent, the results cannot be transitive. Therefore, the transitivity assumption was evaluated by comparing the distribution of potential effect modifiers (publication year, sample size, mean age and percentage male) across studies grouped before analyzing the results.

Assessment of heterogeneity and inconsistency

We use the tau square (τ^2) test and p-value to qualitatively analyze the statistical heterogeneity between the studies. The larger the τ^2 and the smaller the p-value, the greater the possibility of heterogeneity; on the contrary, the smaller the existence heterogeneity. In addition, I^2 is a parameter for quantitative analysis of the heterogeneity between the results of each study. It' s value is distributed from 0-100%. When I^2 is less than 25%, it means that the heterogeneity is low; 25%-50% means that the heterogeneity is moderate; $I^2 > 75\%$ means high heterogeneity. In summary, when $I^2 > 50\%$, it means that there is substantial heterogeneity. We will use global and local methods to test the inconsistency of the research results. For global inconsistency, we evaluated inconsistency statistically using the design-by-treatment test. In addition, we will assessment of local inconsistency by separating indirect from direct evidence (SIDE test) using the R netmeta package. The potential reasons of heterogeneity **(publish year, sample size, mean age, percentage male, BMI, exercise duration, exercise frequency and the total time of single session)** will be explored by network meta-regression.

Publication bias

We compared the adjusted funnel plot to assess the risk of publication bias under specific circumstances. In addition, we made a linear fit for the primary outcome. When the fitted line coincides with the 0 quadrant, it means that there is no published bias. For multi-arm trails, for example, physical activity type A, B and control, we plotted control group vs physical activity type A, and control group and physical activity type B.

Statistical software

The analysis and presentation of results will be performed using the Stata packages network, the R package rjags, gemtc, netmeta, ggplot2, and forestp

Supplementary Information 7: List of available outcomes for included studies

study	exercise program
Turan et al.[13] 2019	yoga
Yüce et al.[14] 2020	pranayama breathing exercise
Thomas et al.[15] 2009	Papworth breathing exercise
França-Pinto et al.[16] 2014	group 1: aerobic exercise pranayama breathing exercise group 2: pranayama breathing exercise
Vempati et al.[17] 2009	yoga
Türk et al.[18] 2002	group 1: high-intensity interval training group 2: high-intensity interval training with Internet based self-management
Ma et al.[19] 2014	aerobic exercise
Malarvizhi et al.[20] 2018	yoga
Bidwell et al.[21] 2012	yoga
Bruton et al.[22] 2017	group 1: DVD and booklet/breathing retraining group 2: Face-to-face intervention/breathing retraining
Silva et al.[23] 2022	group 1: high-intensity interval training group 2: aerobic exercise

Cooper et al.[24] 2003	Buteyko breathing exercises
Duruturk et al.[25] 2018	Inspiratory Muscle Training
Evaristo et al.[26] 2020	group 1: pranayama breathing exercise<br><br>group 2: aerobic exercise
Sarah A. Hiles et al.[27] 2021	yoga
Lage et al.[28] 2021	Inspiratory Muscle Training
Mendes et al.[29] 2010	group 1: pranayama breathing exercise aerobic exercise<br><br>group 2: pranayama breathing exercise
Prem et al.[30] 2012	group 1: buteyko breathing exercise<br><br>group 2: pranayama breathing exercise
Sabina et al.[31] 2005	yoga
Thomas et al.[32] 2012	breathing exercise
Toennesen et al.[33] 2017	group 1: high-intensity interval training<br><br>group 2: high-intensity interval training with diet control
Manocha et al.[34] 2002	yoga
Andreasson et al.[35] 2022	breathing exercise
Zaryyab et al.[36] 2021	group 1: papworth breathing exercise

	group 2:<br>buteyko breathing exercise
Gonçalves RC et al.[37]<br>2008	group 1:<br>pranayama breathing exercise<br>aerobic exercise<br><br>group 2:<br>pranayama breathing exercise
Refaat et al.[38]<br>2015	aerobic exercise<br>resistance training
Scott et al.[39]<br>2013	aerobic exercise<br>resistance training
Holloway et al.[40]<br>2023	papworth breathing exercise
Coulson et al.[41]<br>2021	pranayama breathing exercise
SODHI et al.[42]<br>2009	yoga
SODHI et al.[43]<br>2014	yoga
Cowie et al.[44]<br>2008	buteyko breathing exercises
Agnihotri et al.[45]<br>2018	yoga
Meyer et al.[46]<br>2015	aerobic exercise<br>breathing exercise
Freitas et al.[47]<br>2017	aerobic exercise<br>resistance training

Supplementary Information 8: Risk of Bias

study	Eligibility criteria	Random allocation	Concealed allocation	Baseline comparability	Blind subjects	Blind therapists	Blind assessor	Adequate follow-up dropout \15 %	Intention-to-treat analysis	Between-group comparisons	Point estimates and variability	Score
Turan et al. 2019	1	1	1	1	0	0	0	1	1	1	1	7
Yüce et al.	1	1	1	1	1	1	1	1	1	1	1	10

2020											
Thomas et al. 2009	1	1	1	1	0	0	1	1	1	1	1	9
França-Pinto et al. 2014	1	1	1	1	0	0	1	1	1	1	1	9
Vempati et al. 2009	1	1	0	1	0	0	0	1	1	0	1	6
Türk et al. 2002	1	1	1	1	0	0	0	1	1	1	1	8
Ma et al. 2014	1	1	1	1	0	0	0	1	1	1	1	8
Malarvizhi et al. 2018	1	1	1	1	1	1	1	1	1	1	1	11
Bidwell et al. 2012	1	1	0	1	0	0	0	1	1	1	1	7
Bruton et al. 2017	1	1	1	1	0	1	1	1	1	1	1	10
Silva et al. 2022	1	1	1	1	0	1	1	1	1	0	1	9
Cooper et al. 2003	1	1	1	1	0	0	0	1	1	1	1	8
Duruturk et al. 2018	1	1	1	1	0	0	0	1	1	1	1	8
Evaristo et al. 2020	1	1	1	1	0	0	1	1	1	1	1	9
Sarah A. Hiles et al. 2021	1	1	1	1	0	0	0	1	1	0	1	7
Lage et al. 2021	1	1	1	1	0	1	1	1	1	1	1	10
Mendes et al. 2010	1	1	0	1	0	0	0	1	1	1	1	7
Prem et al. 2012	1	1	1	1	0	0	1	1	1	1	1	9

Sabina et al. 2005	1	1	1	1	1	1	1	1	1	0	1	10
Thomas et al. 2012	1	1	0	1	0	0	1	1	1	0	1	7
Toennesen et al. 2017	1	1	1	1	0	1	0	1	1	1	1	9
Manocha et al. 2002	1	1	1	1	1	1	1	1	1	1	1	11
Andreasson et al. 2022	1	1	0	1	0	0	1	1	1	1	1	8
Zaryyab et al. 2021	0	1	0	0	0	0	0	1	1	1	1	5
Gonçalves et al. 2008	1	1	0	1	0	0	0	1	1	1	1	7
Refaat et al. 2015	1	1	0	1	0	0	0	1	1	0	1	6
Scott et al. 2013	1	1	1	1	0	0	1	1	1	0	1	8
Holloway et al. 2023	1	1	0	1	0	0	1	1	1	1	1	8
Coulson et al. 2021	1	1	0	1	1	0	0	1	1	1	1	8
SODHI et al. 2009	1	1	0	1	0	0	0	1	1	1	1	7
SODHI et al. 2014	1	1	1	1	0	0	0	1	1	1	1	8
Cowie et al. 2008	1	1	1	1	0	0	0	1	1	1	1	8
Agnihotri et al. 2018	1	1	0	1	0	0	0	1	1	1	1	7
Meyer et al. 2015	1	1	0	1	0	0	1	1	1	1	1	8
Freitas et al. 2017	1	1	1	1	0	1	1	1	1	1	1	10

1 eligibility criteria specified, *2* randomization specified, *3* allocation concealment, *4* groups similar at baseline, *5* blinding of assessor; *6* outcome measures assessed in 85% of subjects, *7* intention-to-treat analysis, *8* between group statistical comparisons reported, *9* point measures and measures of variability of outcomes reported, *10* activity monitoring in control groups reported, *11* relative exercise intensity retained constant, *12* exercise volume and energy expenditure.

Supplementary Information 9: Evaluation of heterogeneity and inconsistency

9.1 Quantifying heterogeneity

We use the tau square (τ^2) test and p-value to qualitatively analyze the statistical heterogeneity between the studies. The larger the τ^2 and the smaller the p-value, the greater the possibility of heterogeneity; on the contrary, the smaller the existence heterogeneity. In addition, I^2 is a parameter for quantitative analysis of the heterogeneity between the results of each study. It' s value is distributed from 0-100%. When I^2 is less than 25%, it means that the heterogeneity is low; 25%-50% means that the heterogeneity is moderate; I^2 > 75% means high heterogeneity. In summary, when I^2 > 50%, it means that there is substantial heterogeneity.

outcomes	τ^2	Q	df	P	I^2	Heterogeneity assessment
total scores of QOL	1.2429	941.94	30	<0.0001	96%	high
activity limitations of QOL	1.0594	570.37	24	<0.0001	95.8%	high
emotional functions of QOL	0.6243	339.86	21	<0.0001	93.8%	high
Symptoms of QOL	0.6045	372.92	24	<0.0001	93.6%	high
environmental stimulants of QOL	0.5232	319.29	20	<0.0001	93.7%	high

9.2 Evaluation of inconsistency

Outcomes	the Design-by-Treatment test			
	Q	df	τ^2	p-value
QOL total scores	2.44	6	1.3615	0.8746
activity limitations of QOL	1.72	3	1.2020	0.6336
emotional functions of QOL	4.24	3	0.6258	0.2368
Symptoms of QOL	1.58	3	0.6420	0.6650
environmental stimulants of QOL	4.26	3	0.5269	0.2351

Supplementary Information10: Publication bias

As shown in the figure below, the funnel plot had good symmetry, and the linear fitting line (green) is not perpendicular to the 0 quadrant. Therefore, no small study effect was found for the primary outcome.

10.1 total scores of QOL

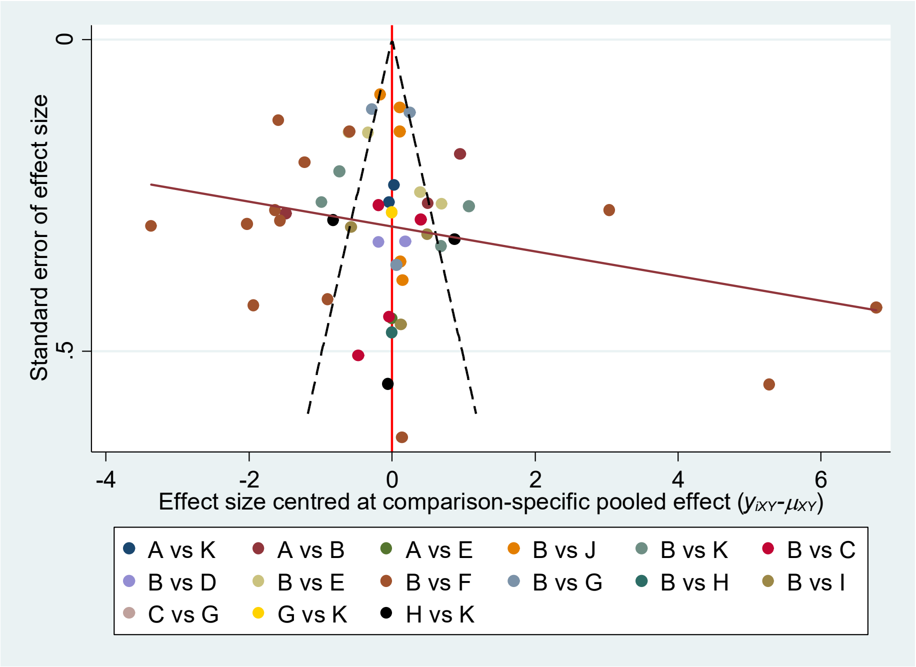

10.2 activity limitations of QOL

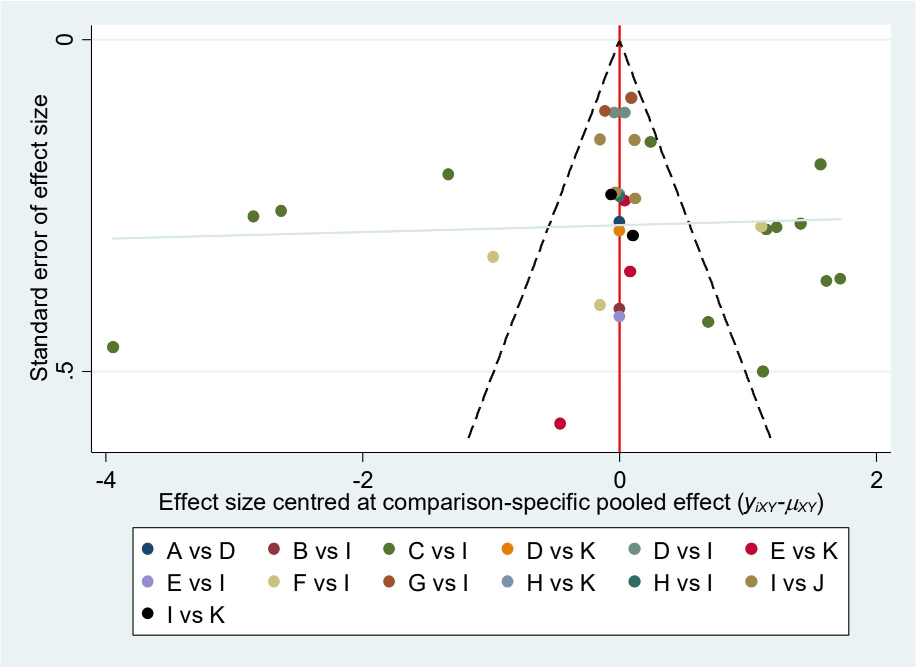

10.3 motional functions of QOL

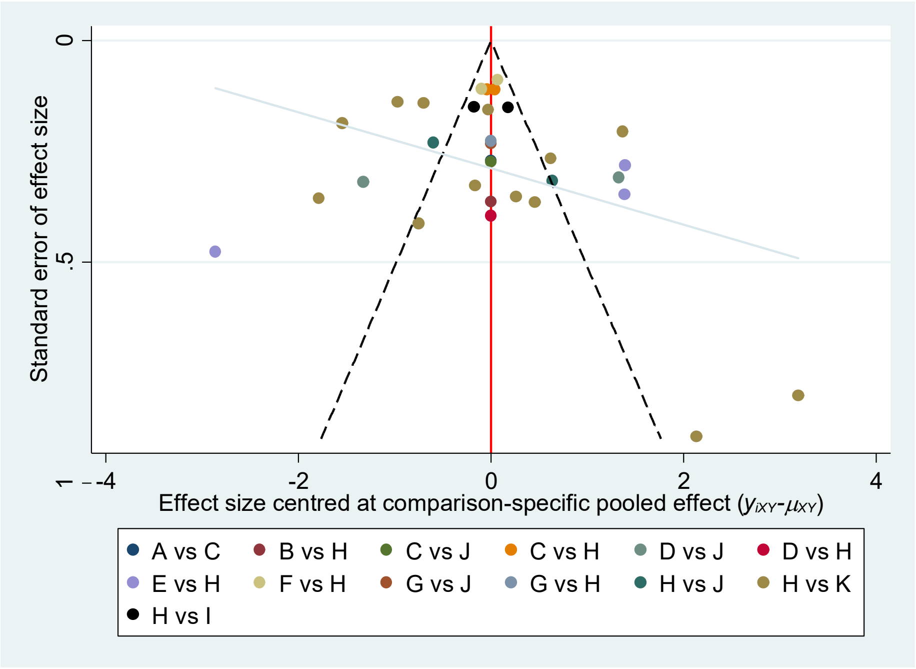

10.4 Symptoms of QOL

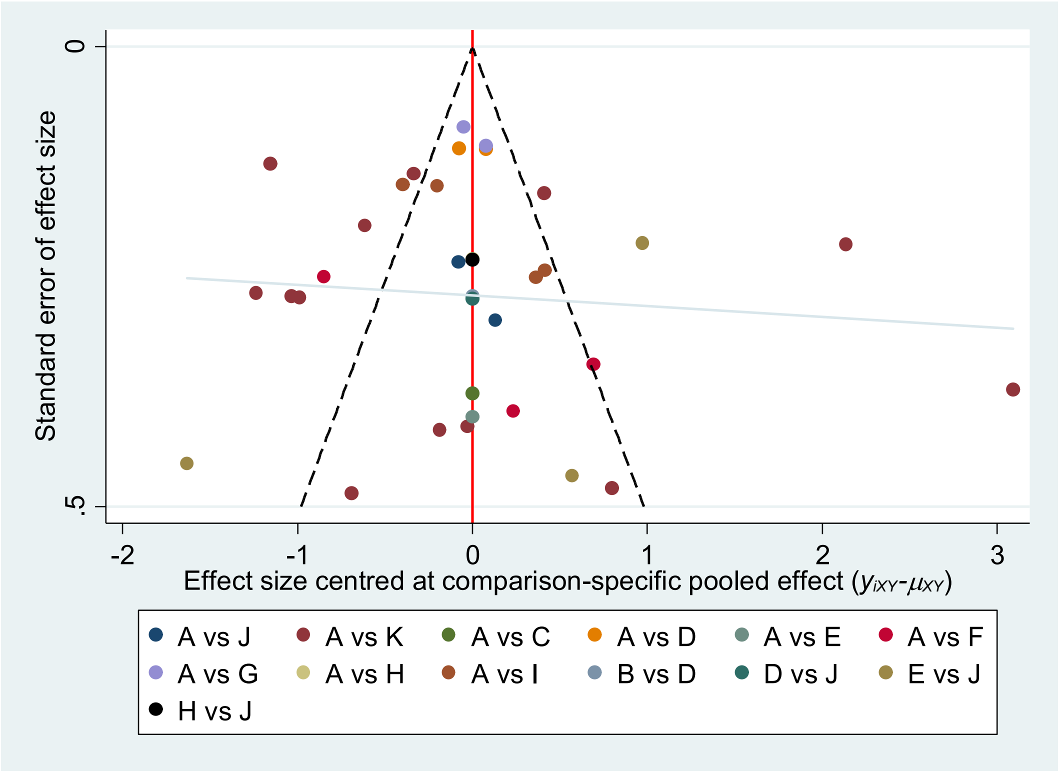

10.5 environmental stimulants of QOL

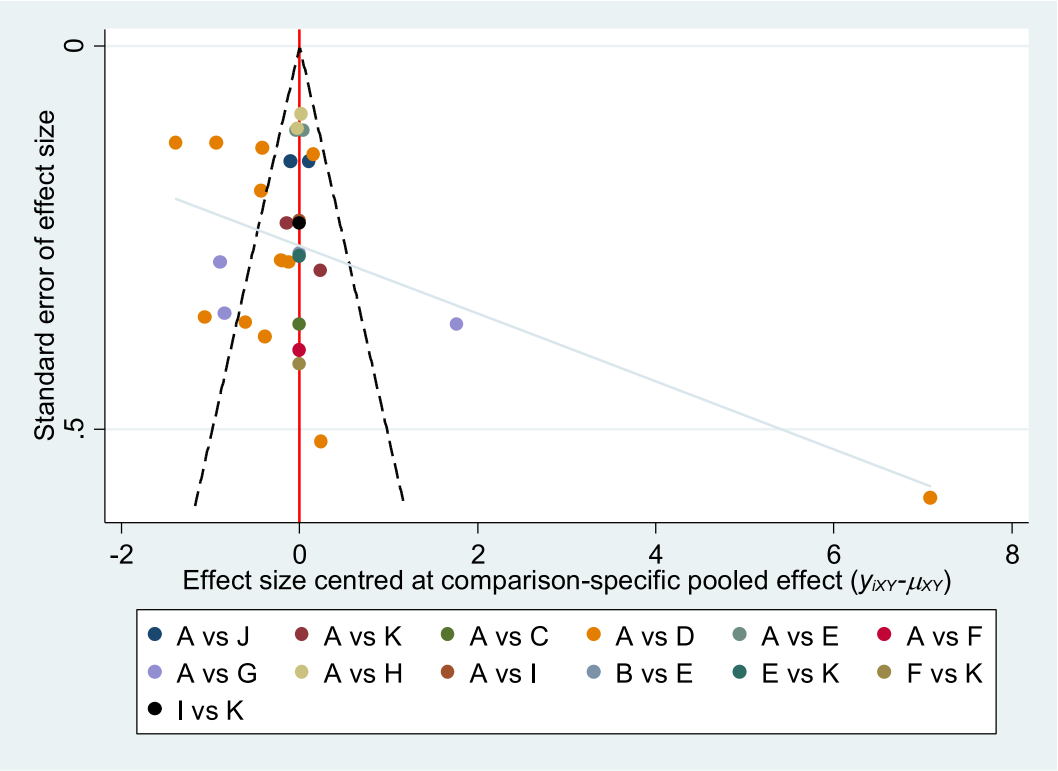

Standard error of effect size
0
.5
-2
0
2
4
6
8
Effect size centred at comparison-specific pooled effect (y_{iXY}-μ_{XY})
A vs J
A vs K
A vs C
A vs D
A vs E
A vs F
A vs G
A vs H
A vs I
B vs E
E vs K
F vs K
I vs K

Supplementary Information 10: Network Meta-Regression

13.1 Changes in heterogeneity: Quality-of-life total Socre

Below we present the results from the changes in heterogeneity in each meta-regression model.

Covariate	Shared beta (median and 95% CrI)	Heterogeneity standard deviation (median and 95% CrI)	% of variance explained
	Quality-of-life		
None	-	0.96 (0.952; 0.966)	-
Publish Year	1.7072 (-0.44595; 3.281)	0.4197 (0.3716; 0.6733)	-29.19%
**Mean Age**	**-3.0968 (-1.7921; -0.5459)***	0.41456 (0.2960; 0.6908)	-29.72%
BMI	-2.0422 (-0.5328; 0.9765)	0.5227 (0.4199; 0.7863)	-19.12%
Percentage Female	1.0634 (-8.7234; 9.981)	0.47764 (0.3609; 0.7276)	-23.27%
**Exercise Period**	**2.9718 (2.28488 ; 3.666)***	0.42489 (0.36743; 0.7287)	-28.63%
**Exercise Frequency**	**-1.21229 (-4.0015; -0.2119)***	0.42451 (0.3973; 0.6909)	-28.67%
**Time of single session**	**-1.5428 (-2.831348; -0.1242)***	0.69032 (0.34678; 0.7906)	-7.29%

CrI: Credible Interval; ⁎: Significant influence factors, 95% CrI does not contain zero.

13.1.1 Publication year

When the model was adjusted for centering value of publish year 2014.109, compared with the control group, the SMD value of physical activity types did not change significantly, and the hierarchy from the unadjusted model retained.

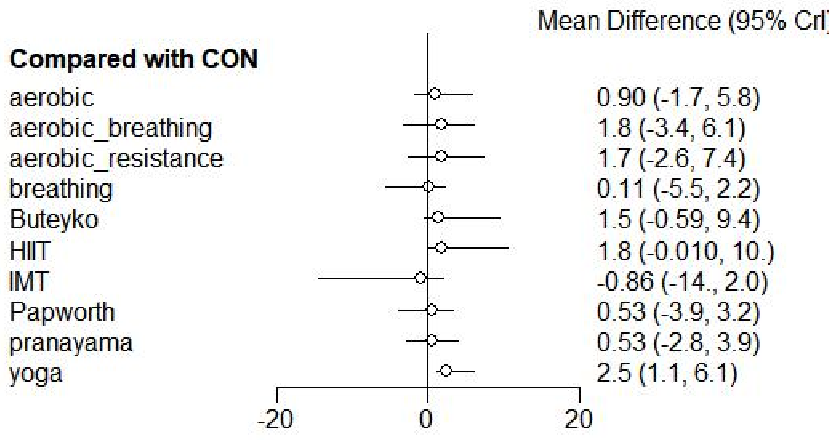

Figure 13.1: Forest plot overall change in motor symptoms adjusted for publish year 2014.109. Physical activity type are ranked according to SMD compared to CON. Treatments crossing the y-axis are not significantly different from CON. *SMD* standardized Mean Difference, *CrI* Credible Interval.

13.2.2 Mean age

When the model was adjusted for centering value of mean age 44.5651, compared with the control group, the SMD value of physical activity types did not change significantly, and the hierarchy from the unadjusted model retained.

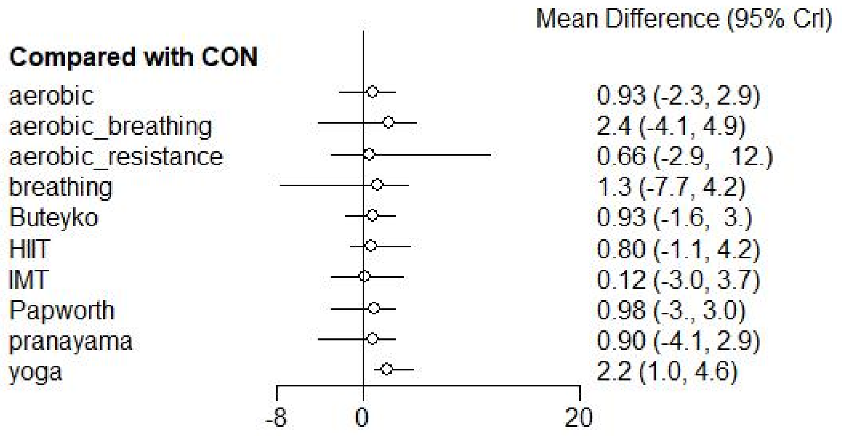

Figure 13.2: Forest plot overall change in motor symptoms adjusted for mean age 44.5651. Physical activity type are ranked according to SMD compared to CON. Treatments crossing the y-axis are not significantly different from CON. *SMD* standardized Mean Difference, *CrI* Credible Interval.

13.2.3 BMI

When the model was adjusted for centering value of BMI 28.03344, compared with the control group, the SMD value of physical activity types did not change significantly, and the hierarchy from the unadjusted model retained.

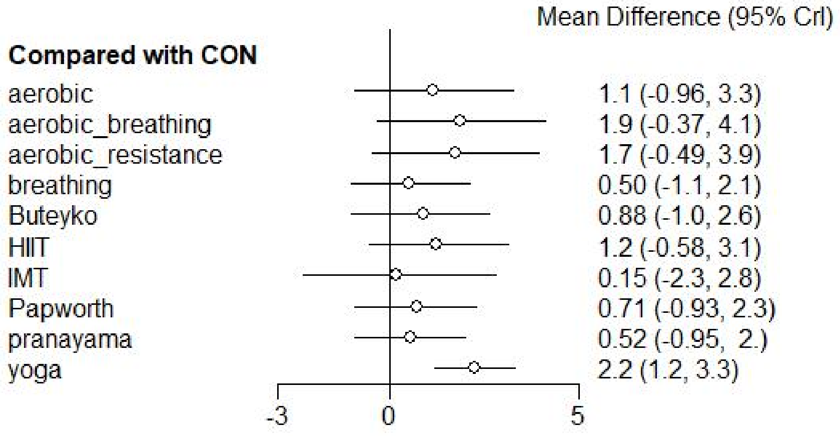

Figure 13.3: Forest plot overall change in motor symptoms adjusted for BMI 28.03344. Physical activity type are ranked according to SMD compared to CON. Treatments crossing the y-axis are not significantly different from CON. *SMD* standardized Mean Difference, *CrI* Credible Interval.

13.2.4 Female percentage

When the model was adjusted for centering value of Female percentage 65.96637, compared with the control group, the SMD value of physical activity types did not change significantly, and the hierarchy from the unadjusted model retained.

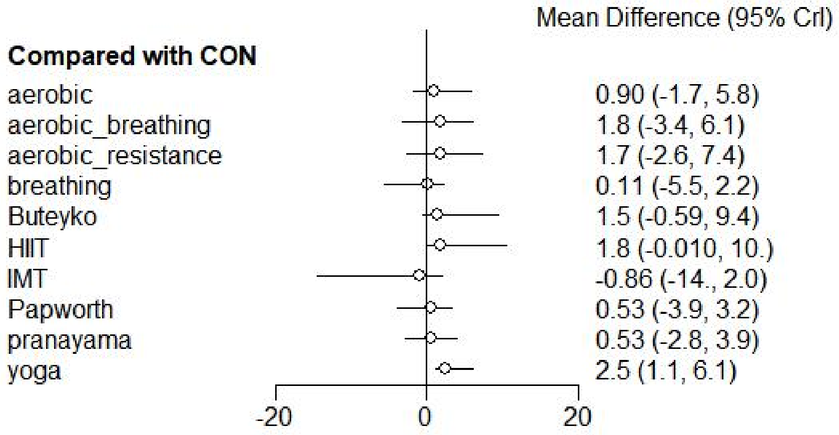

Figure 13.4: Forest plot overall change in motor symptoms adjusted for Female percentage 65.96637. Physical activity type are ranked according to SMD compared to CON. Treatments crossing the y-axis are not significantly different from CON. *SMD* standardized Mean Difference, *CrI* Credible Interval.

13.2.5 Period

When the model was adjusted for centering value of period 10.0456, compared with the control group, the SMD value of physical activity types did not change significantly, and the hierarchy from the unadjusted model retained.

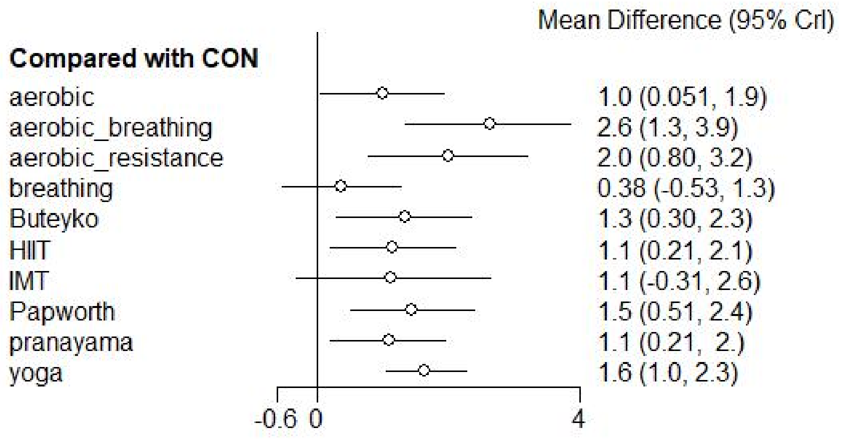

Figure 13.4: Forest plot overall change in motor symptoms adjusted for period 10.0456. Physical activity type are ranked according to SMD compared to CON. Treatments crossing the y-axis are not significantly different from CON. *SMD* standardized Mean Difference, *CrI* Credible Interval.

13.2.6 Exercise Frequency

When the model was adjusted for centering value of exercise frequency 3.891304, compared with the control group, the SMD value of physical activity types did not change significantly, and the hierarchy from the unadjusted model retained.

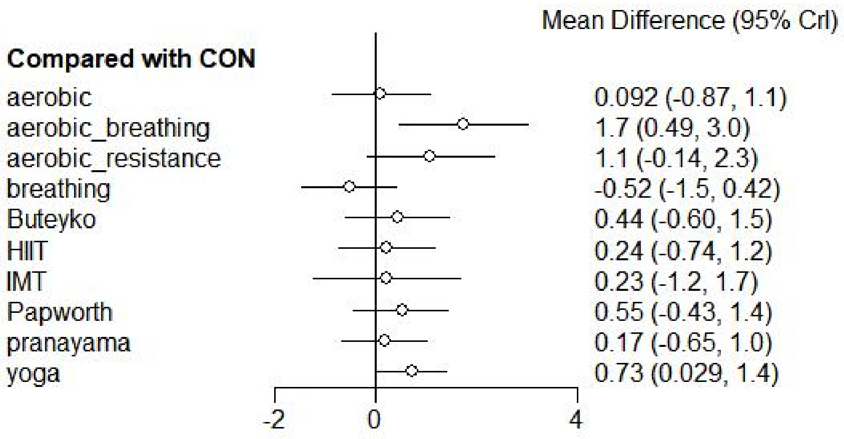

Figure 13.5: Forest plot overall change in motor symptoms adjusted for period 3.891304. Physical activity type are ranked according to SMD compared to CON. Treatments crossing the y-axis are not significantly different from CON. *SMD* standardized Mean Difference, *CrI* Credible Interval.

13.2.7 Time of single session

When the model was adjusted for centering value of time of singlr session 46.84783, compared with the control group, the SMD value of physical activity types did not change significantly, and the hierarchy from the unadjusted model retained.

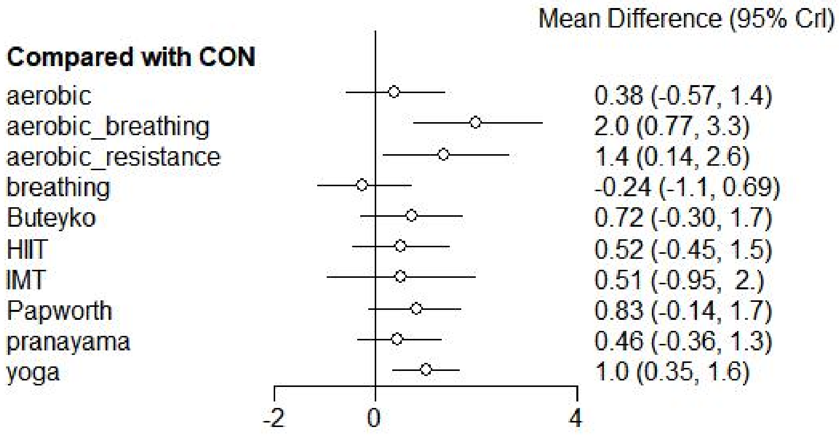

Figure 13.6: Forest plot overall change in motor symptoms adjusted for period 46.84783. Physical activity type are ranked according to SMD compared to CON. Treatments crossing the y-axis are not significantly different from CON. *SMD* standardized Mean Difference, *CrI* Credible Interval.
